# The transport pathway in the ABCG2 protein and its regulation revealed by molecular dynamics simulations

**DOI:** 10.1007/s00018-020-03651-3

**Published:** 2020-09-26

**Authors:** Tamás Nagy, Ágota Tóth, Ágnes Telbisz, Balázs Sarkadi, Hedvig Tordai, Attila Tordai, Tamás Hegedűs

**Affiliations:** 1 Department of Biophysics and Radiation Biology, Semmelweis University, Tuzolto u. 37-47, 1094 Budapest, Hungary; 2 Institute of Enzymology, Research Centre for Natural Sciences, Magyar Tudosok krt. 2, 1117 Budapest, Hungary; 3 Department of Transfusion Medicine, Semmelweis University, Nagyvarad ter 4, 1089 Budapest, Hungary

**Keywords:** ABCG2, Multidrug transport, Molecular dynamics, Cholesterol regulation

## Abstract

Atomic-level structural insight on the human ABCG2 membrane protein, a pharmacologically important transporter, has been recently revealed by several key papers. In spite of the wealth of structural data, the pathway of transmembrane movement for the large variety of structurally different ABCG2 substrates and the physiological lipid regulation of the transporter has not been elucidated. The complex molecular dynamics simulations presented here may provide a breakthrough in understanding the steps of the substrate transport process and its regulation by cholesterol. Our analysis revealed drug binding cavities other than the central binding site and delineated a putative dynamic transport pathway for substrates with variable structures. We found that membrane cholesterol accelerated drug transport by promoting the closure of cytoplasmic protein regions. Since ABCG2 is present in all major biological barriers and drug-metabolizing organs, influences the pharmacokinetics of numerous clinically applied drugs, and plays a key role in uric acid extrusion, this information may significantly promote a reliable prediction of clinically important substrate characteristics and drug-drug interactions.

**Electronic supplementary material:**

The online version of this article (10.1007/s00018-020-03651-3) contains supplementary material, which is available to authorized users.

## Introduction

ABCG2 mediated membrane transport is an important mechanism for the elimination of several toxic substrates from the cell. Localization of this important ABC transporter in all-important tissue barriers (apical surface of hepatocytes, renal cells, blood–brain barrier etc.) and stem cells renders ABCG2 a key factor in drug resistance and uric acid elimination. ABCG2 is also crucial in the pharmacokinetics of several compounds [1–3] which is highlighted by the fact that the US Food and Drug Administration and the European Medicines Agency list ABCG2 among the transporters to be investigated for pharmacokinetics and drug-drug interactions [4, 5]. Experimental data showed that ABCG2 can handle substrates with a wide variety in size and polarity [2, 6, 7]. Uric acid is a small and amphiphilic physiological substrate [8, 9], Hoechts 33342 [10] is a large hydrophobic molecule, and methotrexate [11] or various sulfated and glucuronide conjugates [12] are more polar substrates. Several large molecules, such as Ko143 or elacridar can inhibit substrate transport by a putative competitive inhibition mechanism [13, 14]. A conformation sensitive antibody (5D3), which binds to the extracellular surface of the ABCG2 dimer, also influences the transport cycle and inhibits ATPase activity. Interestingly, this antibody exhibits an increased binding to an inhibitor-bound state of ABCG2 [14–16]. ABCG2 mediated transport is significantly modulated by the lipid composition of the membrane environment [17–19]. ABCG2 transport activity requires cholesterol, but it is not known whether this lipid exerts its effects through affecting the membrane bilayer structure, by binding specifically to the ABCG2 protein as an allosteric modulator, or by both mechanisms [20].

The ABCG2 protein includes an N-terminal intracellular nucleotide-binding domain and a C-terminal transmembrane domain built from six TM helices. The functional form of this half transporter is a homodimer [21, 22]. Cryo-electron microscopy (cryo-EM) studies revealed that the TM helices in ABCG2 are short, bringing the nucleotide-binding domains (NBDs) in close proximity to the intracellular leaflet of the membrane bilayer (Fig. S1). Therefore, the structure of ABCG2 has been classified as a type II exporter fold [18, 23, 24]. The NBD and TMD are connected by a so-called linker region, which involves a V-shaped α-helical region surrounded by highly dynamic segments not resolved in any of the cryo-EM structures [25, 26]. The amphipathic elbow helix, called connecting helix or TH1a in ABCG proteins and the short intracellular loops called coupling helices, play an important role in coupling the conformational changes between the NBDs and TMDs. The large extracellular loop (EL3), which contain intra- and intermolecular disulfide bridges, has been demonstrated to interact with all other extracellular loops and the reentrant, creek-forming G-loop (Fig. S1) [27]. The potential role of the unresolved, highly dynamic segments is currently unknown.

The EL3 loops create a lid-like structure contributing to an exit site to the extracellular space. This lid covers the extracellular ends of TH5 helices involving a so-called Leu-valve (residues 554 and 555) site, which is the boundary of the main drug-binding pocket in the dimer between the TH2, TH2′, TH5, and TH5′. In a cryo-EM study, the estron-3-sulfate substrate was localized in this central, hydrophobic, and cytoplasm-facing cavity [23]. This study demonstrated that N436 and F439 were important residues for substrate recognition and transport. Another cryo-EM study revealed the localization and interaction of MZ29, a Ko143 derivative inhibitor with ABCG2 [18]. The inhibitory molecule was found to fill the central cavity with high affinity and thereby trapping the conformation with separated NBDs. Interestingly, the TMDs were closed in a newly published cryo-EM ABCG2 structure in the absence of ATP, and substrates were shown to open the translocation pathway [28]. Based on an ABCG2 homology model, recently we identified the exit site (Site 4 or cavity 2) and the central binding pocket (Site 3 or cavity 1) using in silico docking of substrates and non-substrates [29]. Moreover, we were able to detect two additional pockets towards the intracellular space. We proposed that Site 2, which involves the position R482, influencing substrate specificity, maybe the site responsible for substrate selection. Site 1 equally interacted with all docked molecules and it was expected to form the entry site of the translocation pathway.

While the highly valuable experimental structures are the fundamentals of structural studies, they inherently lack information on dynamics. Therefore, molecular dynamics simulations have increasingly been used to dissect the effects of mutations on protein structure, expression, and function, as well as to characterize transport mechanism [25, 27, 30]. However, all experimental and the majority of in silico studies focused on a smaller part of the translocation pathway and molecular dynamics simulations with ABCG2 have not involved a substrate molecule. Therefore, in the present study, we performed MD simulations with ABCG2 embedded in a lipid bilayer with the additional presence of a physiological substrate, uric acid. We aimed to characterize the full translocation pathway, highlighting the dynamic alteration of the central drug-binding pocket and the important role of access routes to this binding site.

## Methods

### Structural models

Human ABCG2 structures were used in our simulations in the absence of ATP (PDB IDs: 6HIJ and 6HCO) [18, 24] and in an ATP-bound conformation (PDB ID: 6HZM) [23]. The latter structure was determined in the presence of E211Q mutation to facilitate structure determination by lowering the rate of ATP hydrolysis. We reverted this mutation back to wild type using VMD [31]. Non-protein molecules (e.g. inhibitors and water) were removed. All the structures were trimmed similarly to remove the resolved parts of the linker region and are available for download (https://www.hegelab.org/resources.html).

### Molecular dynamics simulations

Classical molecular dynamics (MD) simulations were used to investigate the effect of cholesterol on ABCG2 dynamics and to characterize the intracellular parts of the substrate translocation pathway. GROMACS 2018 with the CHARMM36m force field was used to run molecular dynamics simulations [32, 33]. Simulations are summarized in Table S1.

Most of the simulation systems were prepared using CHARMM-GUI [34, 35]. First, the structural models were oriented according to the OPM (Orientations of Proteins in Membranes) database [36]. Then all N- and C-termini were patched with ACE (acetyl) and CT3 (N-Methylamide) groups, respectively, and disulfide bridges were set between C592-C608 in chain A, C592′-C608′ in chain B, and C603-C603′ between chains A and B. The 6HIJ structure with associated cholesterol and DOPE (1,2-dioleoyl-sn-glycero-3-phosphoethanolamine) molecules were inserted into a POPC bilayer (1-palmitoyl-2-oleoyl-sn- glycero-3-phosphocholine, *n* = 232). The 6HCO structure was inserted into a pure POPC bilayer (*n* = 228), 1:1 POPC:cholesterol (*n* = 144, *m* = 144), and 1:1 POPC:sitosterol (*n* = 144, *m* = 144). KCl at a concentration of 150 mM was used. Grid information for PME (Particle-Mesh Ewald) electrostatics was generated automatically, and NPT ensemble was selected with a constant number of particles (N), pressure (P) of 1 bar, and temperature of 310 K. Uric acid molecules were inserted into the system by replacing water molecules, using GROMACS tools (e.g. gmx insert-molecules). Parameters for uric acid were generated by the CHARMM general force field (CGenFF) at CHARMM-GUI. In the case of simulations with the ATP-bound 6HZM structure, the protein was inserted into a 1:1 POPC:cholesterol bilayer (*n* = 163, *m* = 163), similarly to simulations with inward-facing ABCG2 structures.

Each system was energy minimized using the steepest descent integrator, which stopped when the largest force in the system becomes less than 500 kJ/mol/nm. To increase sampling, several simulations were forked using the energy minimized system, with different velocities. Six equilibration steps with decreasing position restraints were performed. The corresponding parameter (mdp) files are also available for download. Nose–Hoover thermostat and Parrinello-Rahman barostat with semi-isotropic coupling was employed in the production run. Time constants for the thermostat and the barostat were set to 1 picosecond and 5 picosecond, respectively. Electrostatic interactions were calculated using the fast smooth PME algorithm and LINCS algorithm was used to constrain bonds.

### Targeted molecular dynamics

We used the MOVINGRESTRAINT procedure implemented in PLUMED for transforming the inward-facing apo conformation to an inward-closed, ATP- and substrate-bound conformation. Uric acid was docked to the central binding pocket of the apo structure (PDB ID: 6HCO) using Autodock Vina [37, 38]. Two ATP molecules were placed onto the nucleotide-binding domains in the same conformation as in the ATP-bound structure (PDB ID: 6HZM). To achieve this, isolated Mg-ATP·NBD complexes from the 6HZM structure were aligned to the full-length 6HCO structure and the two Mg-ATP molecules and the apo ABCG2 structure were merged. The Cα and Mg-ATP coordinates were extracted from the ATP-bound 6HZM structure and were used for targeting. The reaction coordinate (collective variable, CV) was the root mean square deviation (RMSD) between the 6HCO and the target 6HZM structures, calculated after an optimal alignment (TYPE = OPTIMAL) of the evolving 6HCO and the static target structures. In the first 5 ns of the simulation, the force constant (κ) was increased from zero to 40,000 kJ/mol/nm, by which the closure of such a large system was achievable as determined by testing several values. In the second 5 ns of the simulations, the force constant was decreased to zero. After this simulation, performed in POPC bilayer, the protein with ATP and uric acid was inserted into a POPC:cholesterol membrane, equilibrated, and used in equilibrium and metadynamics simulations.

### Metadynamics

The translocation through the Leu-valve was accelerated by performing well-tempered metadynamics simulations [39], using GROMACS 2018 in combination with PLUMED [40]. A distance-based collective variable (CV) was biased during metadynamics simulations, namely the distance between the center of masses (COM) of uric acid and four protein Cα atoms (residues 439 and 542) in TH2, TH2′, TH5, and TH5′. To inhibit the backward movement of the substrate towards the intracellular space, a lower wall was defined between the protein COM and the initial position of uric acid (5 Å around protein COM). An upper wall at 38 Å around protein COM was also defined to prevent the distancing of uric acid from the protein. A grid between 0 Å and 40 Å was created and used to speed up calculations. Force at the walls was set to 2,000 kJ/mol/nm. For the biased CV the Gaussian height and sigma were set to 0.6 kJ/mol and 0.06 nm, respectively. The most appropriate width value was calculated from equilibration simulations as the half of the standard deviation of the distance CV. Gaussians were deposited every picosecond. A bias factor of 10 and a temperature of 310 K were set. The same GROMACS options were used as in equilibrium simulations. The time point when uric acid passed the Leu-valve was determined by visual inspection of the six trajectories. PLUMED tools were used for integrating energy (summing the hills).

### Characterization of bilayer properties

100 frames were extracted from each trajectory for analysis. GridMAT-MD [41] was used to calculate bilayer thickness and area per lipid values. The grid was set to 200 and other parameters were the defaults. The following atoms located in the membrane interface at similar z coordinates were set as *atomnames*_*i*_*:* N of POPC, O3 of cholesterol, and O3 of sitosterol. An example parameter file is included for download (https://www.hegelab.org/resources.html).

### Pocket detection

Every 10th frame of simulations with the apo conformation (PDB ID: 6HCO) in POPC:cholesterol bilayer was submitted for mdpocket software [42]. The pockets, which were present at least in 75% of the frames, were extracted from the mdpout_freq_grid.dx grid file, using the extractISOPdb.py script included in mdpocket package.

### Analysis and molecular visualization

Simulations were analyzed by the MDAnalysis Python package [43] and in-house Python scripts (e.g. extraction of cholesterol positions and TH ends). Plots were made by matplotlib Python package [44]. Visualization of structures was performed by PyMOL (The PyMOL Molecular Graphics System, Version 2.0 Schrödinger, LLC.). Specifically, the mobility of cholesterol molecules was characterized by the x and y coordinates of the O3 atom, extracted from the trajectories. Cholesterol molecules located in the extracellular and intracellular membrane leaflets were distinguished by clustering into two groups using the z coordinate of the O3 atom of cholesterol.

### Results

### ABCG2 bound cholesterol molecules promote the closure of TM2 and TM2’

**Fig. 1:**
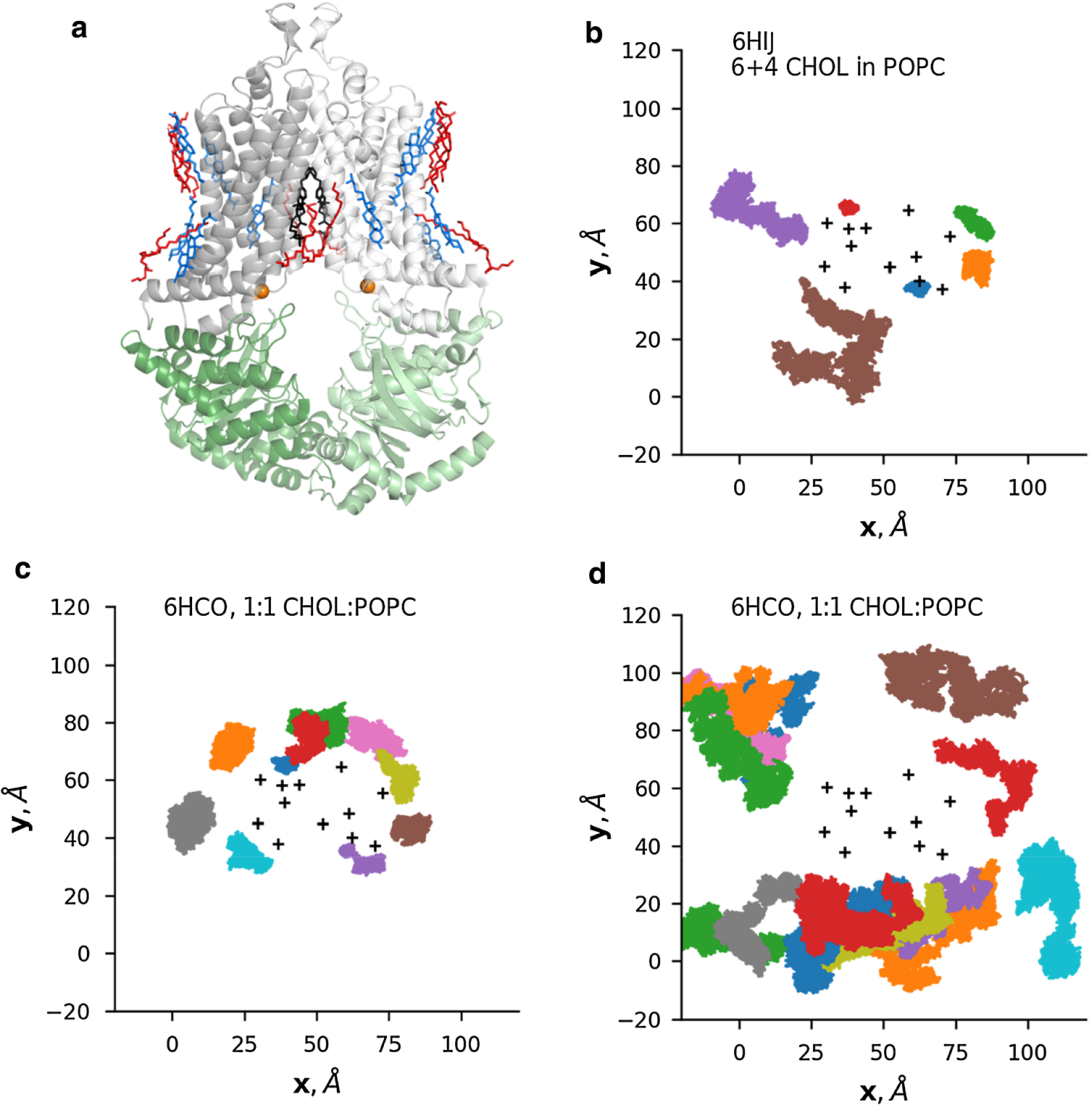
The movement of ABCG2 bound cholesterol molecules is spatially confined. **a** The structure of ABCG2 and associated molecules (PDB ID: 6HIJ). Gray: TM domains, green: nucleotide-binding domains, blue: cholesterol, red: 1,2-Dioleoyl-sn-glycero-3-phosphoethanolamine, black: inhibitor MZ29, orange spheres: Cα atoms of V450 and V450′. **b** The movement of cholesterol molecules in the 6HIJ structure, located in the intracellular membrane leaflet was projected onto the x/y plane. The location of representative cholesterol molecules with more (**c**) and less (**d**) confined motion in a mixed bilayer (1:1 CHOL:POPC) is shown. Black cross ( +) signs: position of TM helices in the starting structure

Several recent ABCG2 structures exist in the RCSB database representing different conformations. Associated lipids (cholesterol and DOPE, 1,2-Dioleoyl-sn-glycero-3-phosphoethanolamine) were also resolved in the case of one cryo-EM map (EMD-4256, PDBID: 6HIJ, Fig. 1) [18]. There is no obvious explanation, why this particular map exhibits immobile lipids molecules since other ABCG2 structures (PDB IDs: 6HCO, 6ETI, 6FEQ, and 6FFC) have also been determined in the same or similar lipid environments, in the absence of nucleotides and the presence of various inhibitors [18, 23]. To gain insight into the interaction between the tightly bound lipid molecules and the ABCG2 protein, we performed equilibrium molecular dynamics simulations using the 6HIJ protein structure. ABCG2 and the associated lipid molecules were inserted into a POPC (1-palmitoyl-2-oleoyl-sn-glycero-3-phosphocholine) bilayer. The movement of the 6 and 4 cholesterol molecules located in the intracellular and extracellular membrane leaflets, respectively, was monitored throughout the 0.5 μs long trajectories (*n* = 3). Projection of their location onto the x/y plane (Fig. 1, Fig. S2) revealed a tight but dynamic interaction. A fraction of the cholesterol molecules remained protein-bound throughout the simulation, while other cholesterol molecules exhibited increased mobility.

Next, we investigated the effect of cholesterol on ABCG2 dynamics. In vivo cholesterol levels of membranes are highly dependent on cell type and organelle and vary between ~ 20 and 50% [45]. To saturate ABCG2 cholesterol sites in our simulations and to avoid limitations set by diffusion in all-atom simulations, we inserted the inward-facing ABCG2 structure (PDBID: 6HCO) into 50–50% POPC-cholesterol, 50–50% POPC-sitosterol, or 100% POPC bilayers using CHARMM-GUI [34, 46, 47]. Sitosterol was used as a control, since this sterol has a different effect on ABCG2 function compared to cholesterol [19, 20]. The initial position of sterol molecules was random, the lipids were evenly distributed, and some of the cholesterol molecules interacting with ABCG2 exhibited restricted motions as in the case of simulations with the 6HIJ system (Fig. 1, Fig. S2). Importantly, both sterols had similar effects on bilayer properties, such as thickness and lipid density (Fig. S3).

**Fig. 2:**
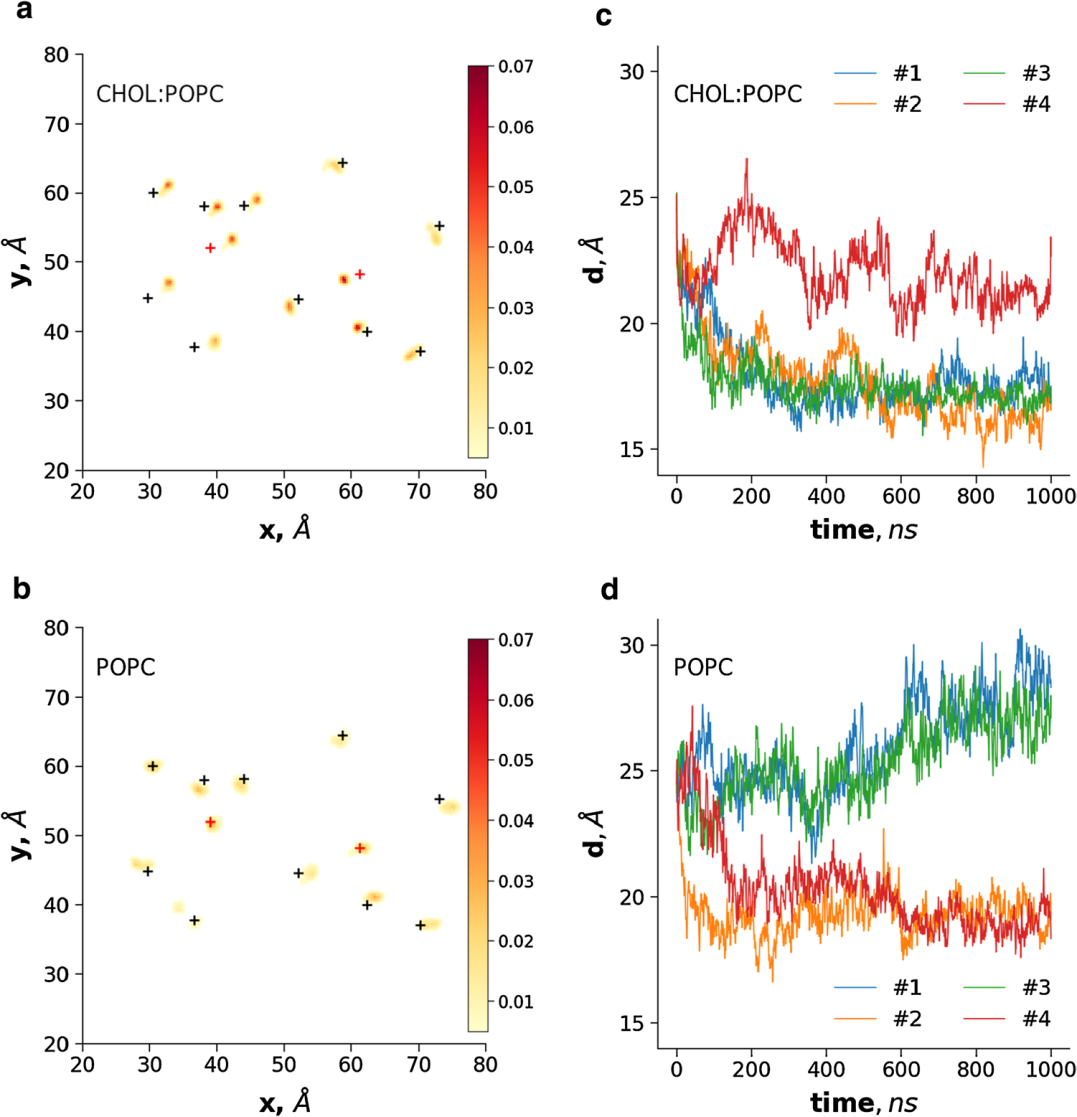
Cholesterol promotes the closure of the intracellular ends of transmembrane helices. Simulations were performed with the inward-facing 6HCO structure in 1:1 cholesterol:POPC mixed (**a**) and POPC-only membrane bilayer (**b**). The x, y coordinates of the Cα atom of the first and last amino acids in each TM helices were recorded in frames after 100 ns. The x and y values were binned and their frequency was plotted in 2D histograms. One out of four simulations is shown for each bilayer. Black crosses: the position of the Cα atoms in the initial structure, red crosses: transmembrane helices 2 and 2′. **c**, **d** The distance between the center of mass of V450 and V450′ Cα atoms, located in the TM2 helices, were plotted

We characterized the mobility of TM helices, which were expected to exhibit altered movements in the presence of cholesterol, in bilayers of different lipid compositions. The position of Cα atoms of amino acids at the boundaries of TM helices (Table S2) was extracted and 2D histograms were calculated and plotted from the x, y components, separately for the distribution of intracellular and extracellular ends. Interestingly, a decreased size of the area visited by the intracellular ends of the inward-facing structure was observed in some of the simulations with cholesterol or sitosterol compared to simulations with a bilayer containing only POPC (Fig. 2, Fig. S4). While in some mixed-lipid simulations the movements of these ends were not so restricted, the bottom part of the helices moved towards the central axis in the presence of cholesterol. Sitosterol also exhibited a restricting effect in one of the simulations, but the ABCG2 TM helices did not move towards the center in a manner observed for cholesterol. The most specific effect of cholesterol was the closure of TM2 and TM2′, characterized by the decreased distance between the intracellular ends of these helices (Cα atoms of V450 from both protomers, Fig. 2). The TM2 movement showed alterations when ABCG2 was placed in pure POPC bilayer. TM2 and TM2′ exhibited either no closure at all or their ends approached each other to a smaller extent than in simulations in the presence of cholesterol. This distance of the intracellular TM helix ends also resulted in fewer interactions between the two NBDs (Fig. S5). It is important to note that POPC tended to infiltrate the TM helices and to interfere with helix closure. The open NBDs of ABCG2 exhibited rigid body motions in these simulations with the apo ABCG2 structure (Fig. S6), and this phenomenon was also observed in simulations with MDR1-like structures in the absence of ATP [48–50].

The above-described cholesterol effects can be observed for the intracellular but not for the extracellular helix ends (Fig. S4), where transmembrane helices are tightly packed. The differences vanish in the case of the inward-closed structure with transmembrane helices tightly packed at both sides (Fig. S4 and Fig. S6).

### Drug access and binding pocket volumes are affected by dynamic fluctuations

**Fig. 3:**
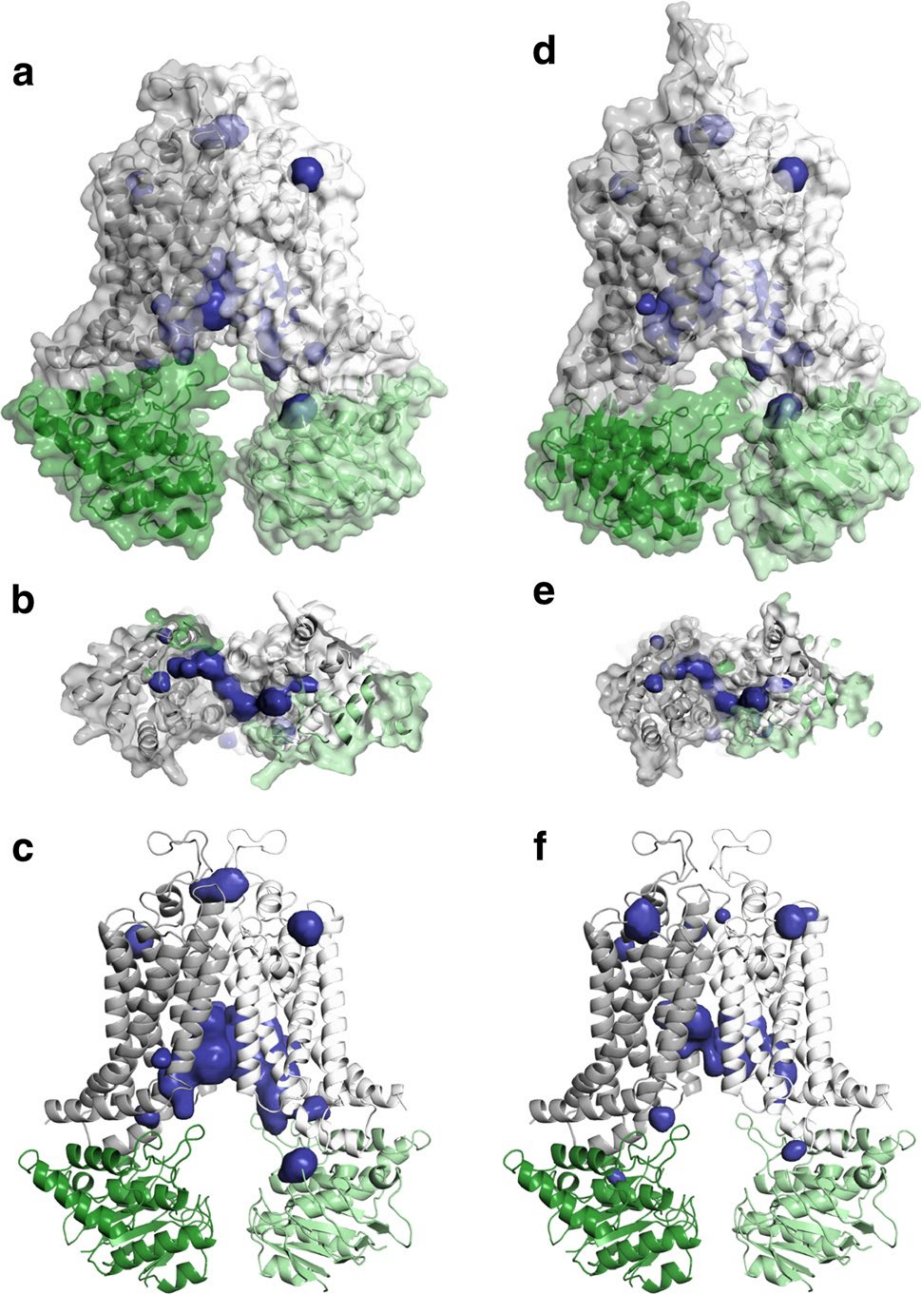
ABCG2 drug binding cavities in MD simulations correspond to pockets identified by in silico docking. **a–c** Binding pockets determined in an MD trajectory are shown in the context of the starting structure. **d**, **e** The closure of the NBD and TM helices limits access to the translocation pathway. **f** In some of the simulations, spaces forming a translocation pathway are less frequent and less continuous. Gray and white: transmembrane domains, green and light green: nucleotide-binding domains, blue: pockets

To test the effect of conformational fluctuations on the internal cavities sufficient for drug binding, pockets were identified through trajectories using the MDpocket software [42]. Identified cavities present with a frequency of 0.75 or more were analyzed and are shown in the context of the initial structure (Fig. 3a–c). In two simulations a large cavity involving Site 2, Site 2′, and Site 3 was observed. This complex cavity was exposed both to the membrane bilayer (Fig. 3b) and towards the cytosol (Fig. 3c). However, the cytosolic TM2 closure prevented the lateral opening (Fig. 3d) and also limited the access to the central cavity (Site 3) from the cytosol (Fig. 3e). In some of the parallel simulations exhibiting a similar degree of closure, the slightly different conformation of TM helices resulted in smaller drug binding cavities (Fig. 3f).

### Equilibrium simulations reveal substrate engagement along the translocation pathway

**Fig. 4:**
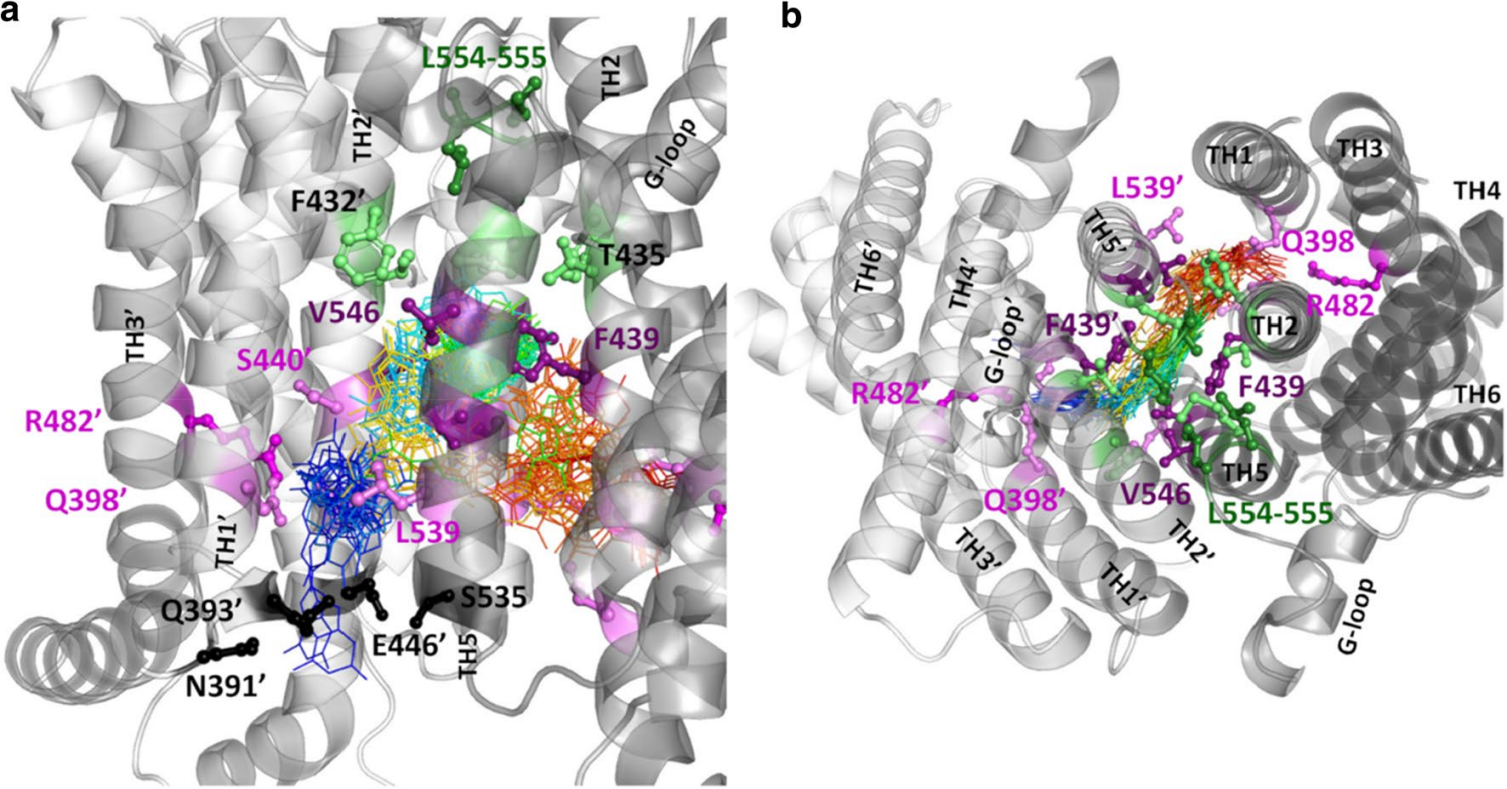
The movements of the substrate, uric acid along the translocation pathway. In an equilibrium simulation with the inward-facing ABCG2 structure (PDBID: 6HCO) in the presence of uric acid molecules, a single uric acid molecule entered Site 2′ (a pocket between TM1, TM2, and TM3 in chain B, magenta and violet sticks) from the cytosolic region (black sticks). Uric acid moved forward from Site 2′ (R482′) to Site 3 between TM2, TM2′, TM5, and TM5′, finally to Site 2 at the opposite side in chain A. Line colors of blue-green-yellow–red correspond to uric acid and encode increasing simulation time points; deep purple: N436, F439, T542, and V546; light green: F432, T435, T538, and M549; green: Leu-valve, gray: transmembrane helices; **a** side view; **b** top view from the extracellular space

To discover the details of substrate translocation, we performed equilibrium simulations with the inward-facing ABCG2 structure (PDBID: 6HCO) embedded in a POPC/cholesterol bilayer in the presence of a substrate. We used uric acid, which is a physiological substance transported by ABCG2 [8, 9]. Since our simulation time scale was relatively short (0.5–1 µs), we increased the possibility of substrate binding and translocation by incorporating 30 uric acid molecules in the simulations (Fig. 4).

During one of the 0.5 µs long simulations, the distance between the cytoplasmic sides of the TM helices decreased to an extent that abolished the substrate entry point. During another 0.5 µs long simulation, a single uric acid molecule out of 30 entered Site 2′ (a pocket between TM1, TM2, and TM3 in chain B) from the large space between the partially separated NBDs via an entry point surrounded by amino acids N391′, Q393′, E446′, and S535 (Fig. 4). The substrate interacted with amino acids Q398′, S440′, S443′, R482′, and L539 with the highest frequency in Site 2′ and moved forward, towards the extracellular space to Site 3 between TM2, TM2′, TM5, and TM5′. The most frequent substrate interaction partners included amino acids F439, T542, and V546 from both protomers and also N436 with a lower frequency. The uric acid molecule also moved further towards the extracellular space interacting with F432, T435, and M549 from both transporter halves, but did not reach the Leu-valve (residues 554 and 555). In the final phase, uric acid changed its moving direction towards the intracellular space and reached Site 2 in chain A at the opposite side of its entry point. The most frequent interactions (Q398, S440, S443, R482, and L539′) corresponded to the same residues observed at the opposite Site 2’.

### The critical translocation step through the Leu-valve is characterized by metadynamics

While the backward movement of uric acid towards the intracellular space was partly inhibited by the closure of the intracellular TM helix ends, the substrate did not move into the extracellular space. This was expected since the inward-facing conformation after closing motions is still more open compared to known ATP-bound conformations. Therefore, we placed two Mg-ATP molecules to the nucleotide-binding domains and docked a single uric acid molecule into Site 3, and used a targeted MD protocol for “morphing” this inward-facing conformation with ligands into the inward-closed, 6HZM-like conformation (Fig. S7). The protocol was driven by minimizing the difference (RMSD) between the simulated 6HCO complex and the static, inward-closed conformation. This ATP- and substrate-bound model was used in 1 µs long simulations to test the exit of uric acid. During one of the simulations, the substrate moved towards the intracellular space and escaped in the direction of the central axis between TM3, TM3′, TM5, and TM5′ (not shown). During two additional simulations, the substrate moved further towards the extracellular space and on a single occasion, it also moved down to Site 2, as in the bottom open conformation discussed above (Fig. 4). Since we were unable to observe the final step of substrate transport to the extracellular space, we set up accelerated simulations using metadynamics.

**Fig. 5:**
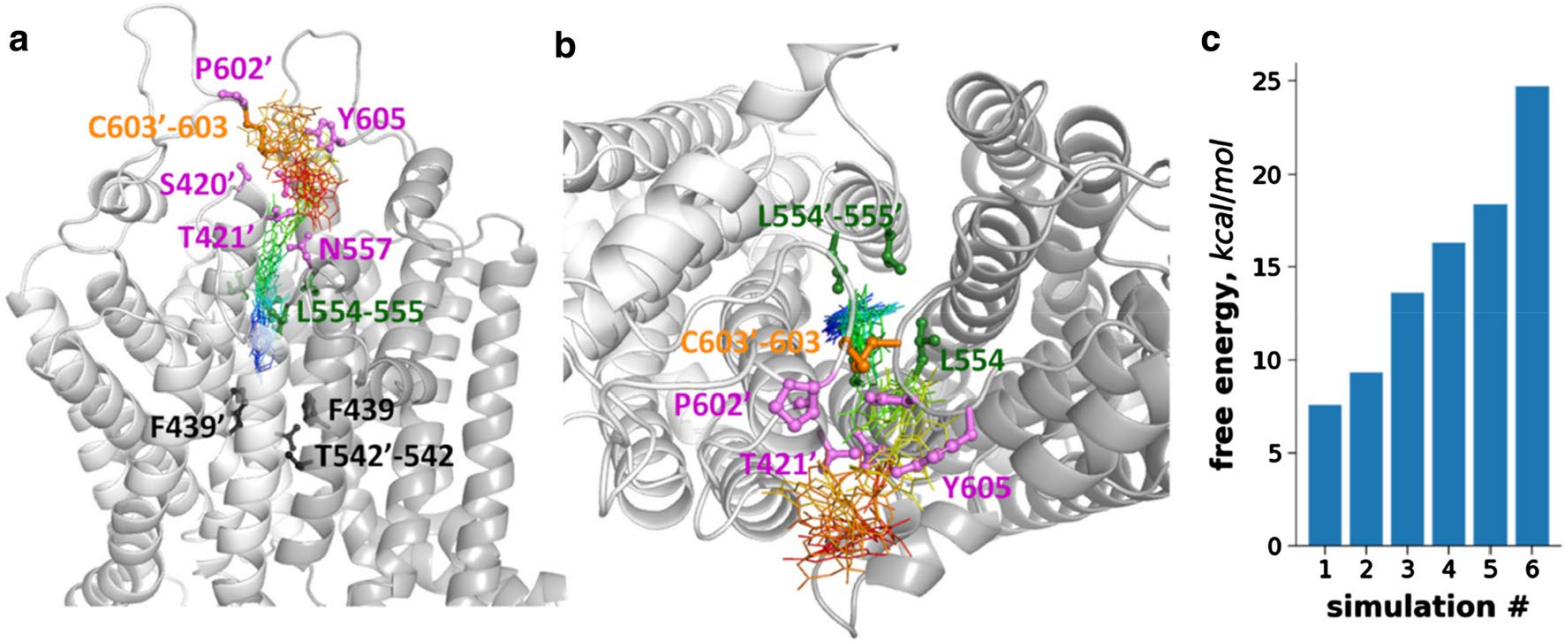
The transit through the Leu-valve as characterized by metadynamics. **a**, **b** A 6HZM-like structure generated by a targeted MD simulation with two ATP molecules and a uric acid in Site 3 was subjected to metadynamics simulations. The resultant conformation was used in six metadynamics simulations with a reaction coordinate defined by the distance between the center of mass of Cα atoms of four amino acids (F439, T542 from both protomers, black sticks) and the center of mass of the uric acid molecule. Blue-green-yellow–red lines represent uric acid and correspond to increasing simulation time points. Uric acid passed through the Leu-valve (green sticks) in all simulations. Amino acids in the extracellular gate are labeled with violet. **c** Biasing energy accumulated until the time point, when the uric acid molecule passed the Leu-valve

Since convergence was not achieved in longer metadynamics simulations due to the inability of the substrate to find the path back to the central pocket, we were unable to calculate the free energy surface of the substrate export. Therefore, we aimed to perform several short simulations to the point when uric acid passed the Leu-valve and to calculate the energy barrier associated with each trajectory. We ran six metadynamics simulations with a reaction coordinate (collection variable, CV) defined by the distance between the center of mass of Cα of F439, F439′, T542, and T542′ and the center of mass of the uric acid molecule (Fig. 5 and Fig. S8). During metadynamics simulations, the CV was calculated at regular intervals (here, every picosecond) and a positive Gaussian potential is added to the energy at the current position in the energy landscape of the system. This increasing potential in time and along the CV discourage uric acid to stay at the same location, thus the small-molecule substrate was expected to move to other positions. We set a lower wall of 5 Å for this distance CV to inhibit exit towards the intracellular space. We also set an upper wall of 38 Å to restrict the substrate in a volume close to the protein that permits the sampling of the CV space between 5 Å and 38 Å. During all of the simulations, the uric acid molecule passed the Leu-valve and then left the intra-protein space (Site 4) after some lateral movements, since the space above the Leu-valve was limited by the lid formed by the extracellular loops. The substrate interacted with residues N601′, P602′ from one protomer and residues N604, Y605 from the other. The amino acids around the exit included 556–559, 616–618, and the extracellular end of the opposite TM2 (residues T421 and N425). To gain insights into the energetics of the substrate passage, we summed the hills of the bias up to the time point when the uric acid molecule passed the Leu-valve (Fig. 5c). In three out of six simulations the free energy input for passing the leucine amino acids was between 7–13 kcal/mole, which is comparable to the energy liberated in association to the hydrolysis of an ATP molecule. It is important to note that we could not consider an important degree of freedom, the conformation freedom of the protein, for biasing in metadynamics that results in large differences in the height of the energy barriers.

## Discussion

Earlier, we described the effect of mutations (e.g. Q141K and R482G) on ABCG2 dynamics and identified potential hot spots along a drug translocation pathway using a homology model [29]. Since there were only small differences between our homology model and the first published experimental ABCG2 structure [24], most of our results and conclusions are still valid. However, several important questions remained unanswered and motivated our current work. The most challenging issue was how to reconcile experimental data on cholesterol binding motifs and a static structural model, as we did not perform simulations in the presence of cholesterol in our previous work. Here, we performed simulations in the presence of sitosterol and cholesterol, which sterols promoted an increased ABCG2 transport function in wet experiments to different levels. We observed an increasing probability and extent of closure of the cytoplasmic ends of TM helices as a consequence of lipid environment changes. In pure POPC, the distance between TH2 and TH2′ decreased to a value close to 20 Å in two out of four simulations (Fig. 2). In the presence of sitosterol, the closure could be observed to a similar level as in pure POPC bilayers (Fig. S4). However, in the absence of cholesterol, lipids tended to infiltrate the helices compared to simulations in the presence of cholesterol. During some of the simulations, both sterols decreased the lateral movement of TM helices as expected as a consequence of altered bilayer properties, such as the increased order of lipid chains (Fig. S3) [51–53]. Our results strongly suggest that cholesterol limits the conformational space of ABCG2 and facilitate the closure of the TM helices. In addition, the effect of cholesterol is likely not to be exclusively attributed to the physical changes of the bilayer, since both sitosterol and cholesterol had similar general effects on membrane properties in our simulations (Fig. S3). Ordered cholesterol molecules at specific sites were observed in a cryo-EM density (PDB ID: 6HIJ), and these lipids remained bound also for a longer period at those sites during MD simulations. Thus further studies are required to elucidate the effects of lipids on ABCG2, similar to the in silico investigations performed by Sansom et al*.* [54–56] for other membrane proteins.

Regarding the substrate translocation pathway within the ABCG2 protein, the present MD simulations reinforce the presence of a pathway from Site 1 to Site 4, described in our former in silico docking study. The existence of the central Site 3 in this pathway has been confirmed by several cryo-EM structures with the bound substrate and inhibitor molecules. In addition to structural studies, amino acids indispensable for substrate binding and transport have been identified in this site, using various biochemical methods [23, 57]. Surprisingly, Gose *et al.* highlighted F439 as a single residue necessary for engaging transport [57]. The transport of small molecules used in their study was affected by mutations at position F439 but not at position N436. In another study by Manolaridis *et al*. [23], mutating the latter position was reported to abolish the export of a different molecule, estron-3-sulphate. Importantly, the small uric acid molecule used in our study contacted F439 with the highest frequency, and several other residues, including N436 with moderate frequency.

Another thorough experimental work supplemented with molecular dynamics simulations addressed the interactions of the longest extracellular loop (EL3) and the role of leucine amino acids at the constriction between Site 3 and Site 4 [27]. It was demonstrated that EL3 constrained by disulfide bonds interacts with the reentrant G-loop and all extracellular loops forming a lid to cover the upper cavity, thus creating Site 4 (off-site). Based on molecular dynamics simulations in the absence of any substrate molecule, they proposed that transport between the two sites is driven by the squeezing motion of leucine 555 residues, indicating a peristaltic transport mechanism [58, 59].

By metadynamics simulations (Fig. 5), here we demonstrate that the passage of a small substrate molecule, uric acid through the Leu-valve requires energy not greater than the hydrolysis of an ATP molecule. Importantly, this does not mean that moving a single substrate molecule requires one ATP, only indicates that a relatively small amount of energy is able to drive the substrate through the valve in some of our simulations. Interestingly, the inward-closed conformation is regarded as a structure developed after transport, since the substrate, which was included during the structure determination process (PDB ID:6HZM) [23], was not present in the constricted binding pocket of this structure. Therefore, the low energy required for substrate passage without biasing the protein conformation in our simulations strongly suggests the absence of large energy differences between the pre- and post-transport steps, further supporting a transport mechanism involving peristaltic motions with rather small conformational changes.

The entry of substrates from the intracellular direction to the central binding pocket has not been studied yet. The general view is that the inward-facing conformation provides unlimited access to the central binding pocket from the bottom. Even if this was the case, a molecule would not necessarily diffuse into the binding pocket without interactions with other residues along its path. To observe the entry of a substrate into the pocket we performed equilibrium simulations (no extra force applied) when uric acid molecules were also present. Surprisingly, a uric acid molecule did not immediately enter the central binding pocket, but first visited Site 2′ involving R482. Even after uric acid reached the central pocket and remained close to the crucial F439 residue, the substrate turned backward but this time became located to Site 2 at the opposite half transporter polypeptide chain. These additional interactions further support that the R482-site is likely to be an important location along the translocation pathway. Although we characterized the first steps of substrate transport, the initial step of substrate entry and possibly the substrate recognition process are likely to be more complex. First of all, the small uric acid molecule did not enter directly the central binding site, since it is possible that the gate was not sufficiently open at the critical time point when the uric acid started its penetration into the protein. In addition, the unresolved flexible intracellular loop regions not present during our simulations are likely to have an effect on substrate entry. The tip of the loop between the first and second NBD β-strands is not resolved in cryo-EM structures and may act as a gating helix analogous to structurally similar regions in bacterial transporters [60–62]. This role is supported by mutations, which are located in this loop and affect substrate specificity (Fig. S9). Importantly, the recent apo-closed ABCG2 structure strongly suggests that the substrates have a profound role in opening up the translocation pathway [28].

In summary, our results presented here emphasize that substrate specificity is unlikely to be defined by a single central binding pocket but several additional sites are involved along the translocation pathways. In our opinion, multiple pathways may exist, which should be imagined not as several channels through the protein, but as one or more potential channels with many translocation patterns, similar to a route with several alternative handles on a climbing wall. Along a single route, climbers with different heights or different finger strengths may use different handles. Similarly, in the case of ABCG2, one substrate may enter into Site 2 and interact with R482, and as a consequence, its transport will be influenced by R482 mutations. Another substrate may not fully enter Site 2 or may enter but not interact with R482, thus the transport of this substrate will be unaffected in the presence of the R482G variation. For similar reasons, N439 is likely to be important for E1S transport but not for some other substrates as discussed above. While the C55S amino acid substitution does not alter the transport of SN-38, further experiments with other substrates may highlight the influence of this residue on substrate specificity. Even the proposed multiple ABCG2 drug binding pockets [63] may not represent different drug-binding pockets in a classical sense, but alternative interaction surfaces along the translocation pathway with different affinities for various substrates. In conclusion, we propose that interactions of small molecules with ABCG2 shall be characterized along their translocation pathway to predict the transport behavior of different substrates. Moving towards the paradigm of utilizing translocation pathways or patterns instead of single drug binding pockets may potentially lead to more efficient and higher validity predictions of drug-drug interactions.

### Electronic supplementary material

Below is the link to the electronic supplementary material.

Supplementary file1 (PDF 5687 kb)

## Data Availability

Input structures and parameter files can be downloaded from https://www.hegelab.org/resources.html
