# Supplementary material for: The transport pathway in the ABCG2 protein and its regulation revealed by molecular dynamics simulations
Source: Cell Mol Life Sci. 2020 Sep 26;78(5):2329–39. doi: 10.1007/s00018-020-03651-3 (PMC7966132; doi:10.1007/s00018-020-03651-3)
Supplement: Supplementary file 1 — Supplementary file1 (PDF 5687 kb) [file 18_2020_3651_MOESM1_ESM.pdf]

SUPPLEMENTARY METERIAL

A full transport pathway within the ABCG2 protein is revealed by molecular dynamics simulations

Tamás Nagy, Ágota Tóth, Ágnes Telbisz, Balázs Sarkadi, Hedvig Tordai, Attila Tordai, Tamás Hegedűs

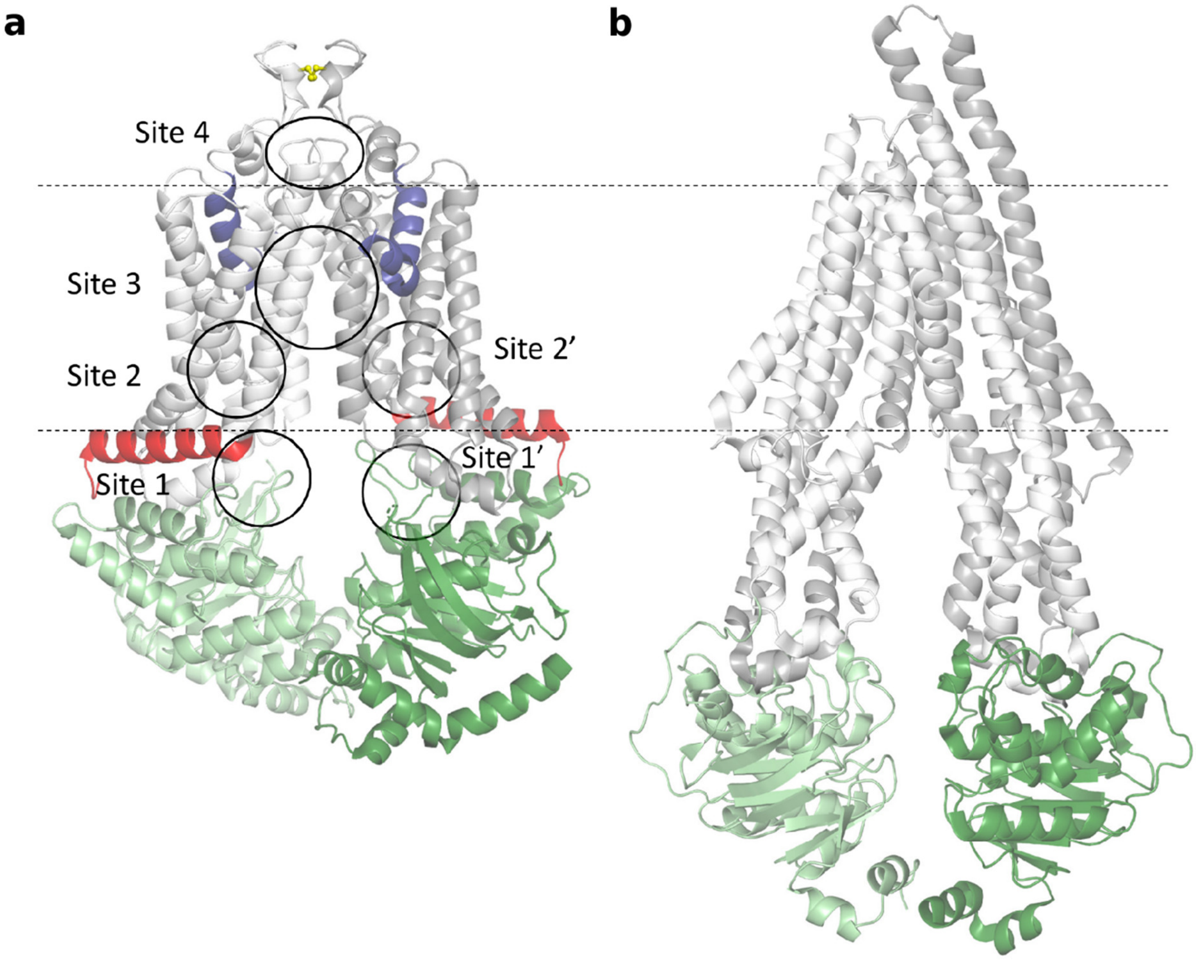

supp_figure_typeIIstruct. Structural organization of ABCG2 and comparisons to that of MDR1. (**a**) The nucleotide binding domains (green colors) are located close to the membrane bilayer (dashed lines indicate the approximate location relative to the membrane bilayer) in type II exporters, such as ABCG2 (PDB ID: 6HIJ). (**b**) In contrast, the long intracellular extensions of TM helices in MDR1 (PDB ID: 6QEX), which possesses a type I exporter fold, mandates a larger distance of NBDs from the cell membrane. Yellow: intermolecular disulfide bond between Cys603 and Cys603'; blue: reentrant G-helix (reentrant from the extracellular space); red: connecting helix. Circles indicate potential drug binding sites, which have been identified by *in silico* docking [1]. Site 1: entry site, Site 2: R482 site, Site 3: central binding pocket, Site 4: exit site.

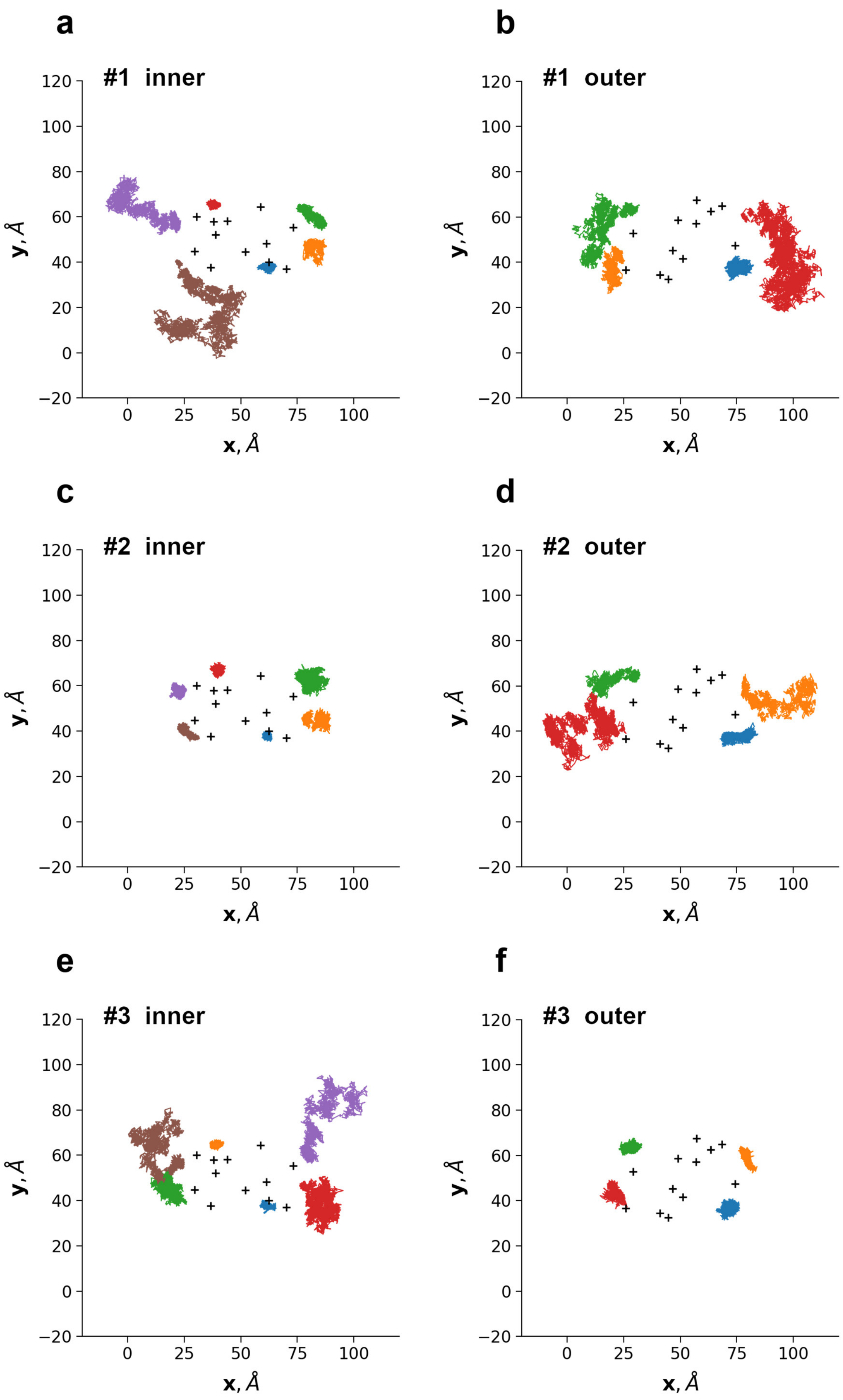
a
#1 inner
b
#1 outer
c
#2 inner
d
#2 outer
e
#3 inner
f
#3 outer
y, Å
x, Å
120
100
80
60
40
20
0
−20
0
25
50
75
100

supp_fig_chol1. The movement of ABCG2 bound cholesterol molecules is confined in both the extracellular and intracellular bilayer leaflets. The movement of the six and four cholesterol molecules, which were observed in the cryo-EM map [2], were extracted from MD simulations performed with the corresponding ABCG2 structure (PDB ID: 6HIJ, n=3) and projected onto the x/y plane.

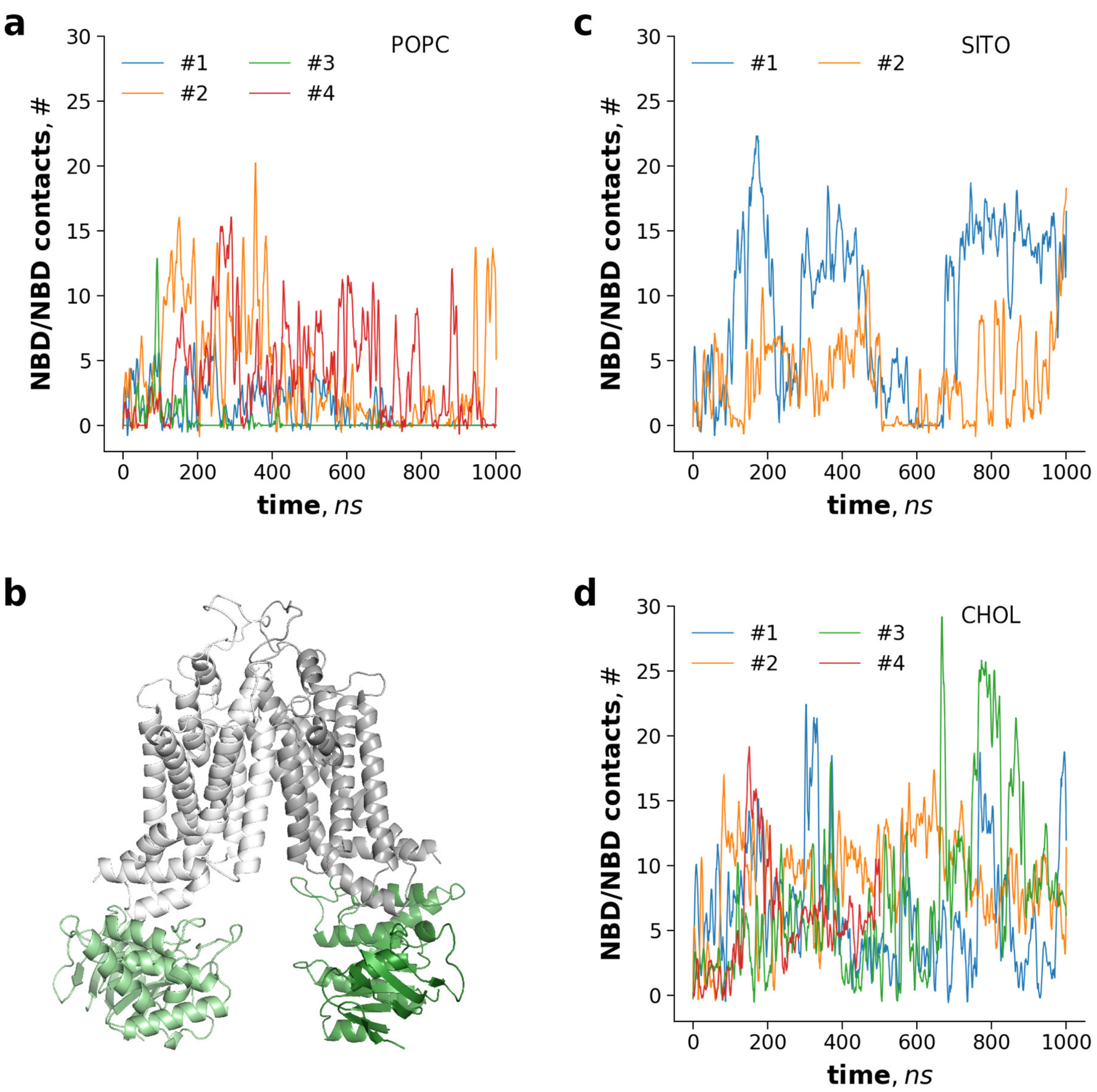

supp_fig_nbdcontacts. Nucleotide binding domains are more separated in pure POPC membrane. In simulations using the inward-facing conformation of Type I ABC folds, the closing of the separated nucleotide binding domains can be observed [3-5]. Since a similar closing motion may influence the studies of substrate entry and translocation, we characterized the dynamics of the inward-facing ABCG2 conformation by calculating the contacts between the two NBDs over the trajectories. (**a, b**) The number of contacts between NBDs were low or even became permanently zero in simulations in a bilayer built only from POPC. The large separation is maintained by infiltration of lipid molecules between TM helices. (**c**) NBDs in a POPC:sitosterol bilayer dissociate completely at a lower frequency compared to POPC only bilayers. (**d**) In contrast, NBDs are almost always in contact in cholesterol containing membrane. The large fluctuations are the consequence of the rigid body motions of NBDs (supp_fig_rmsd). Contact are defined if the distance of Cα atoms in two amino acids does not exceed 7.5 Å and were extracted using MDAnalysis. NBD: nucleotide binding domain, POPC: 1-palmitoyl-2-oleoyl-sn-glycero-3-phosphocholine.

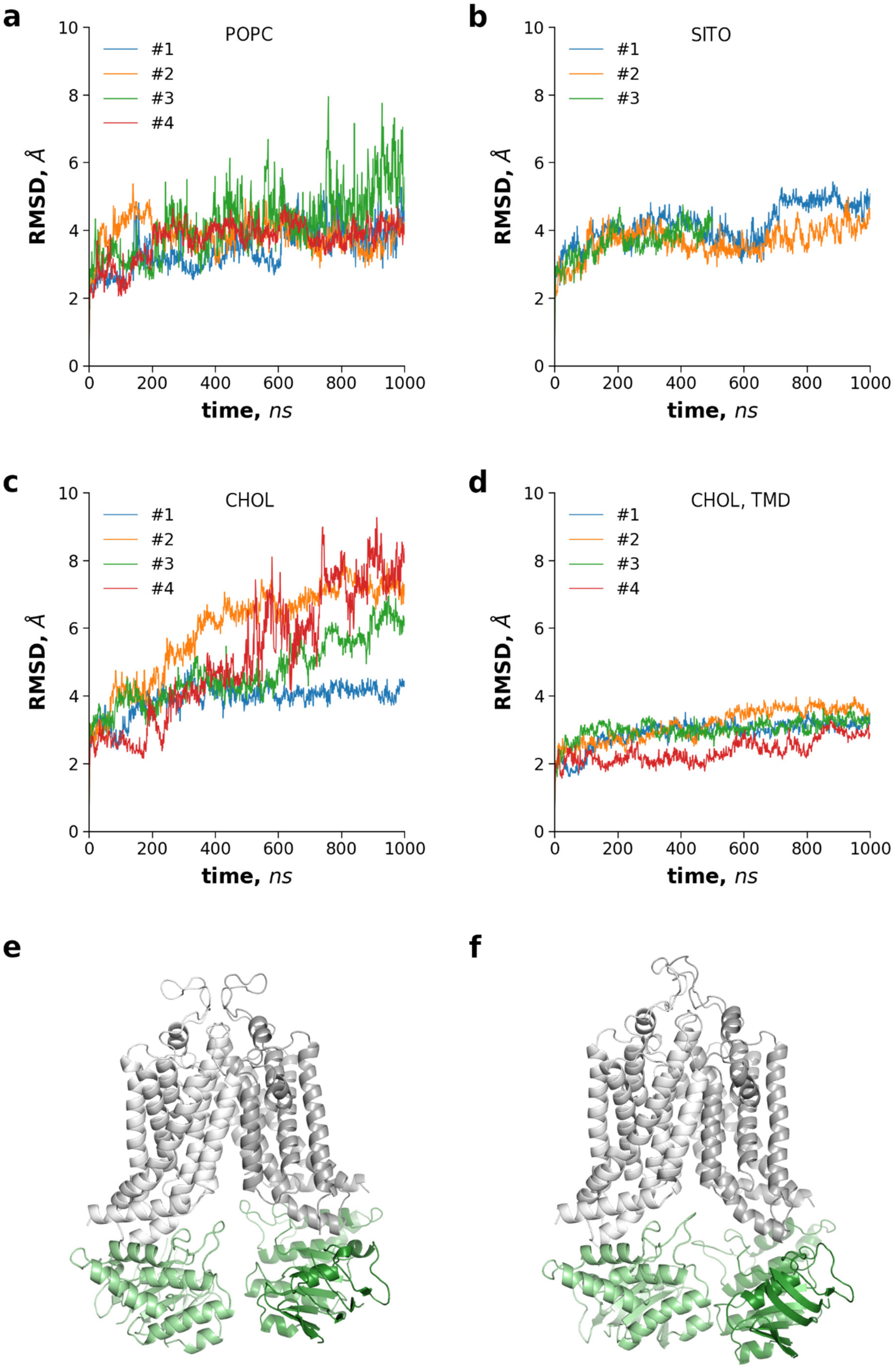
a
POPC
#1
#2
#3
#4
RMSD, Å
time, ns
b
SITO
#1
#2
#3
RMSD, Å
time, ns
c
CHOL
#1
#2
#3
#4
RMSD, Å
time, ns
d
CHOL, TMD
#1
#2
#3
#4
RMSD, Å
time, ns
e
f

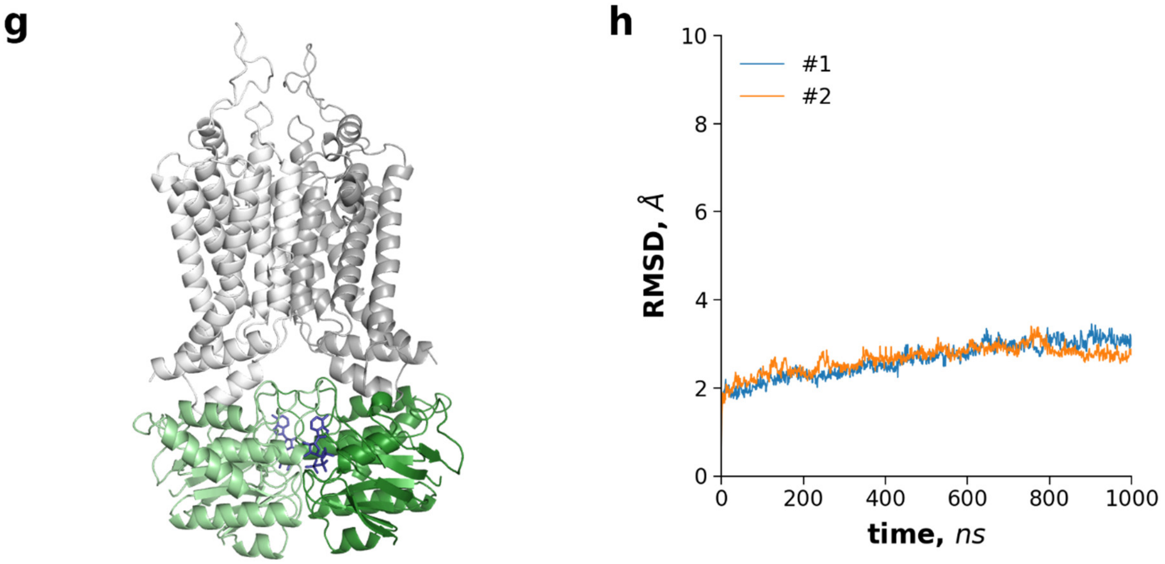

supp_fig_rmsd. The inward-facing ABCG2 structure exhibited mobile nucleotide binding domains. The root mean square deviation (RMSD) of apo structure (PDBID:6HCO) from the initial conformation was moderate in POPC **(a)** and POPC:sitosterol (**b**) containing membrane bilayers. (**c**) The larger changes were mainly contributed to rigid body motion of the NBDs. In contrast, the RMSD was high in a POPC:cholesterol bilayer. (**d**) These elevated RMSD values were also caused by the rigid body motion of the NBDs as demonstrated by the low RMSD values calculated only for the TMDs. (**e**) Compared to the initial structure, in one of the simulations as an example, one of the NBDs slightly moved towards the central axis and the bilayer. A similar NBD motion was also observed in a previous study in the presence of ATP [6]. (**f**) In our case, the absence of ATP likely causes unmasking of the repulsive interactions between NBDs and the rolling of the other NBD away that can be dissected in future studies similar to simulations by Szollosi *et al.* [7]. The inward-closed, ATP-bound structure (PDBID: 6HZM [8]) is also tightly packed at the extracellular side (**g**) that results in increased intramolecular interactions and restricted dynamics (**h**).

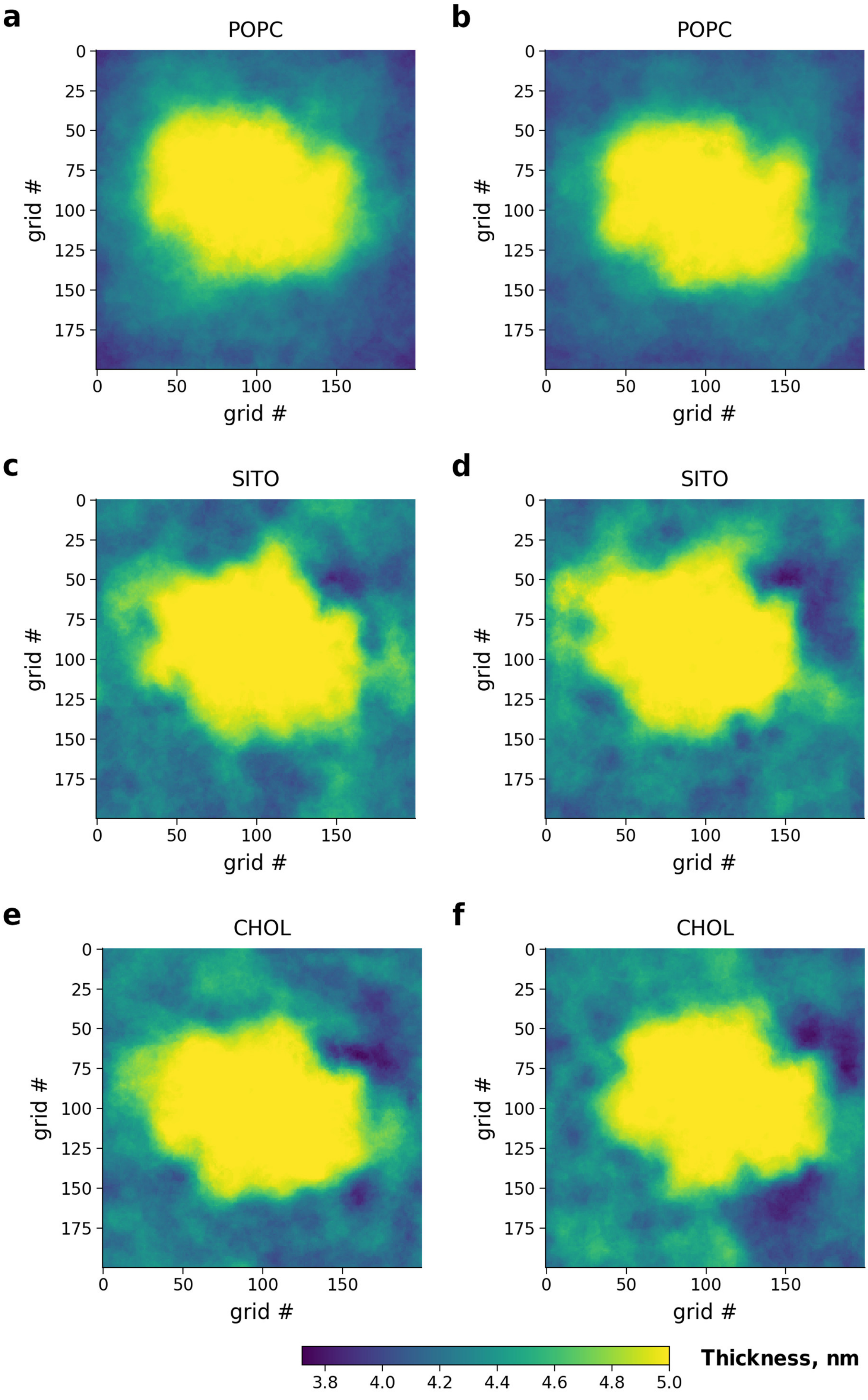
a
POPC
b
POPC
c
SITO
d
SITO
e
CHOL
f
CHOL
grid #
0
25
50
75
100
125
150
175
0
50
100
150
Thickness, nm
3.8
4.0
4.2
4.4
4.6
4.8
5.0

g

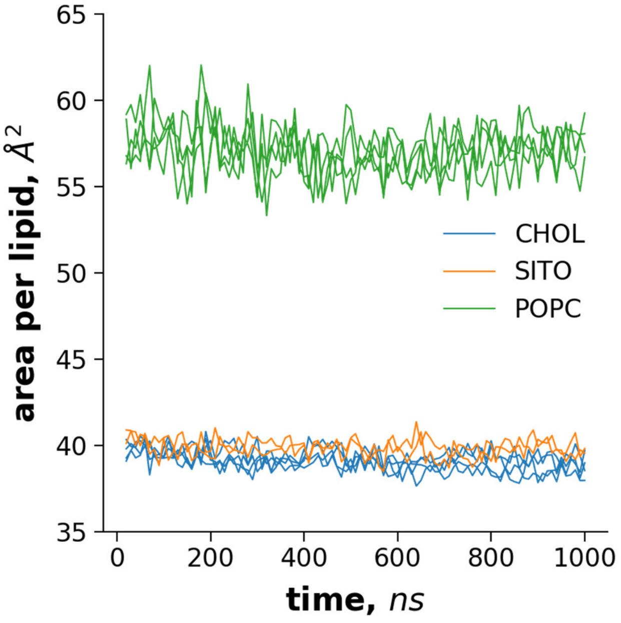

supp_fig_bilayerprop. Lipid composition dependent membrane bilayer properties. (a-f) Membrane thickness of bilayers were calculated using the GridMAT-MD software [9] for simulations with the inward-facing structure (PDB ID: 6HIJ) in different bilayers. Pure POPC membrane exhibited a smaller thickness and a more even thickness distribution than mixed bilayers. Density plots are shown for two independent simulations. Large yellow area: protein. Thickness and area per lipid values were calculated for 100 frames and averaged. **(g)** Area per lipid was calculated also with the GridMAT-MD software. Lipid molecules in sterol containing membranes required smaller space, as expected for cholesterol (CHOL) or sitosterol (SITO) containing membranes because of increased spatial order of lipid tails.

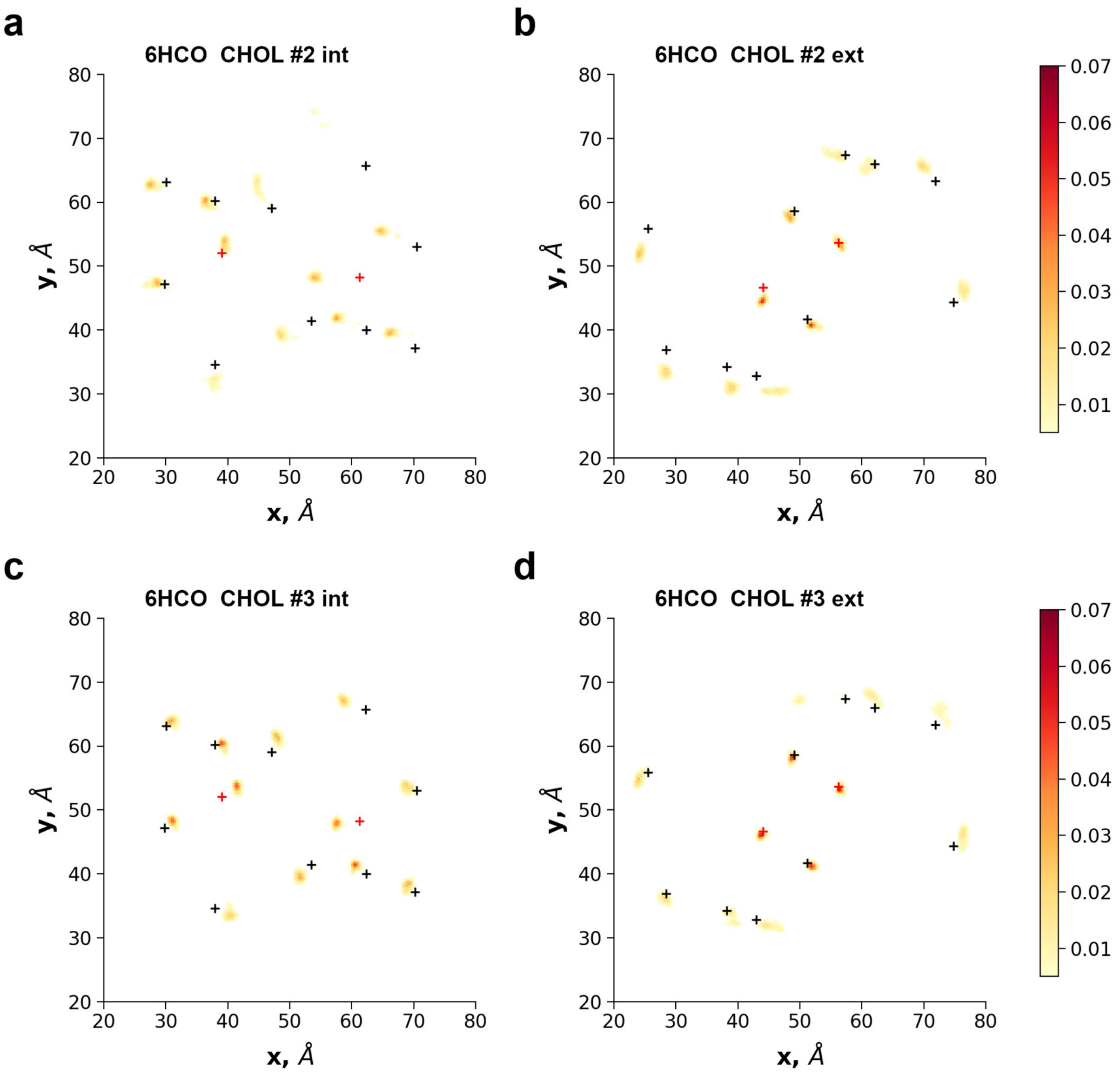
a
6HCO CHOL #2 int
b
6HCO CHOL #2 ext
c
6HCO CHOL #3 int
d
6HCO CHOL #3 ext
x, Å
y, Å
20
30
40
50
60
70
80
0.01
0.02
0.03
0.04
0.05
0.06
0.07

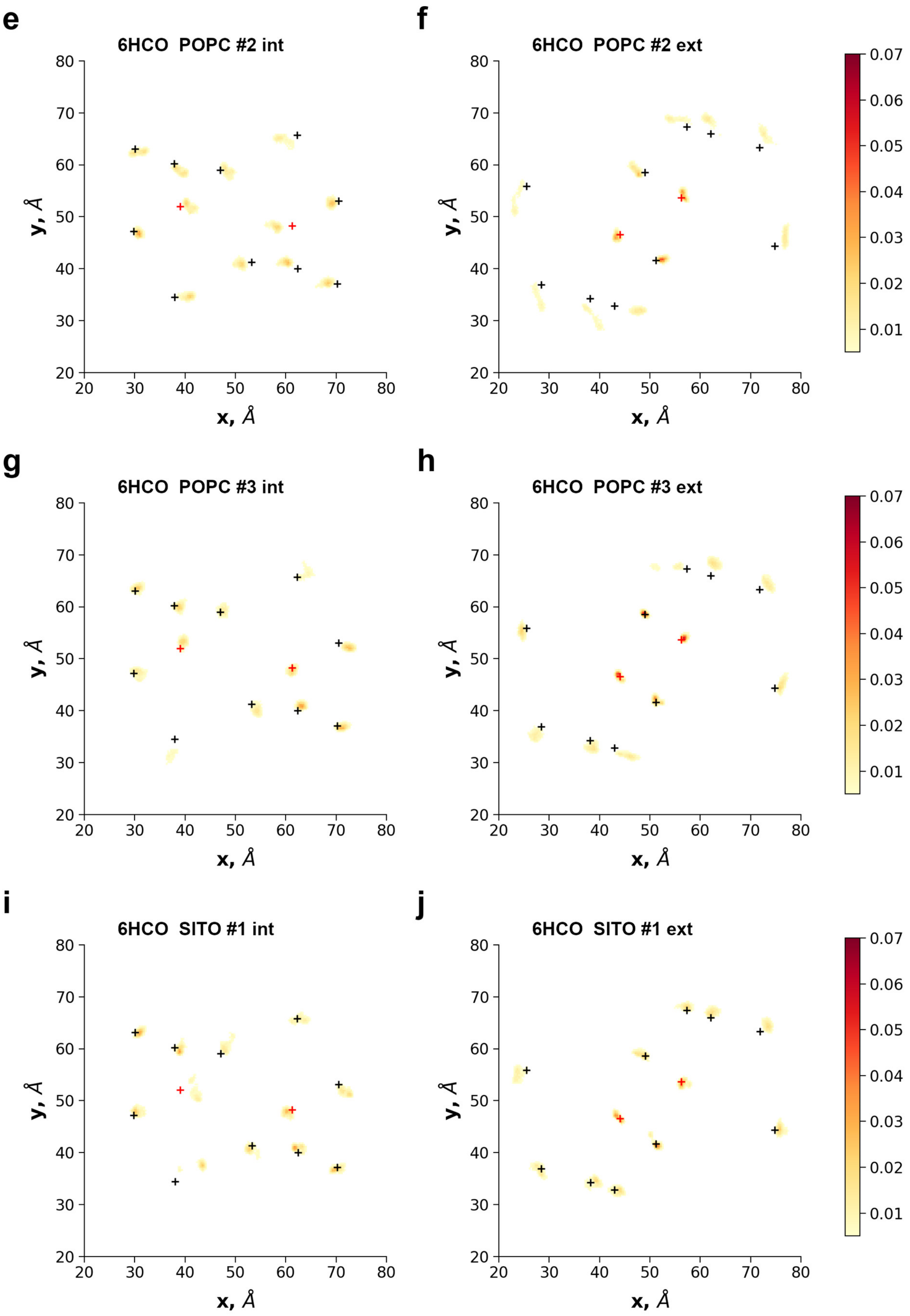
e
6HCO POPC #2 int
f
6HCO POPC #2 ext
g
6HCO POPC #3 int
h
6HCO POPC #3 ext
i
6HCO SITO #1 int
j
6HCO SITO #1 ext
y, Å
x, Å
20
30
40
50
60
70
80
0.01
0.02
0.03
0.04
0.05
0.06
0.07

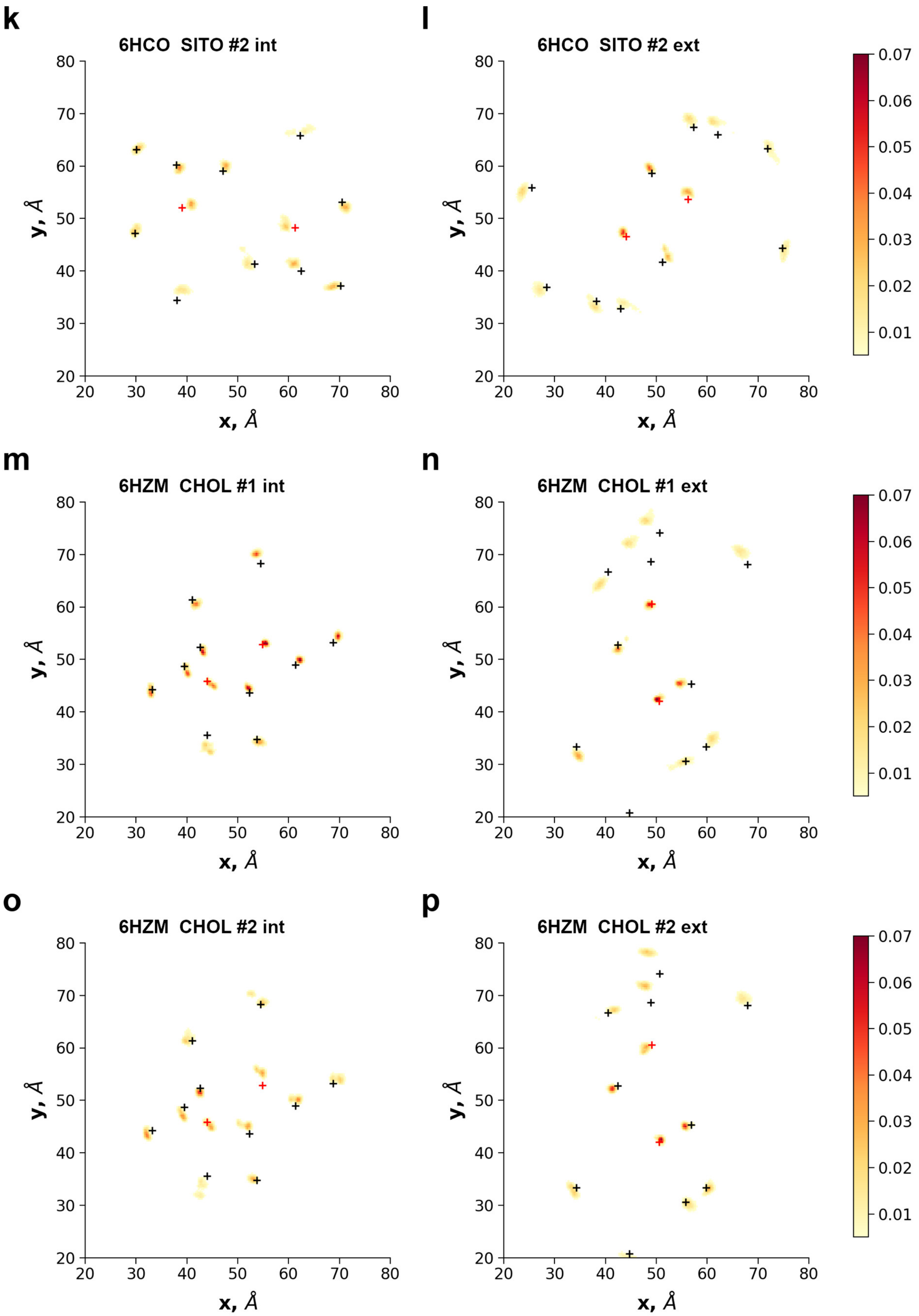
k
6HCO SITO #2 int
l
6HCO SITO #2 ext
m
6HZM CHOL #1 int
n
6HZM CHOL #1 ext
o
6HZM CHOL #2 int
p
6HZM CHOL #2 ext
y, Å
x, Å
0.07
0.06
0.05
0.04
0.03
0.02
0.01

supp_fig_chol2. Cholesterol promotes the closure of the intracellular ends of transmembrane helices of the inward-facing structure. The position of Cα atoms of amino acids at the boundaries of TM helices was extracted and 2D histograms were calculated and plotted from the x, y components, separately for the distribution of intracellular (int) and extracellular (ext) ends ("+" markers, red: TH2). Plots were generated from two independent simulations for each system as examples. The bottom open conformation (PDBID: 6HCO) in CHOL:POPC (**a-d**), pure POPC (**e-h**), and CHOL:SITO (**i-l**) membrane bilayers. The Cα of amino acids at positions 450 (TH2) is labeled with red. Since the extracellular ends of the helices are more tightly packed than the intracellular ends and thus are more stable, lipid composition dependent changes at the outer side were not expected in our simulations. (**m-p**) A similar phenomenon is observed in simulations with the ATP-bound ABCG2 structure (PDBID: 6HZM), which exhibit tightly packed helices both at intracellular and extracellular sides.

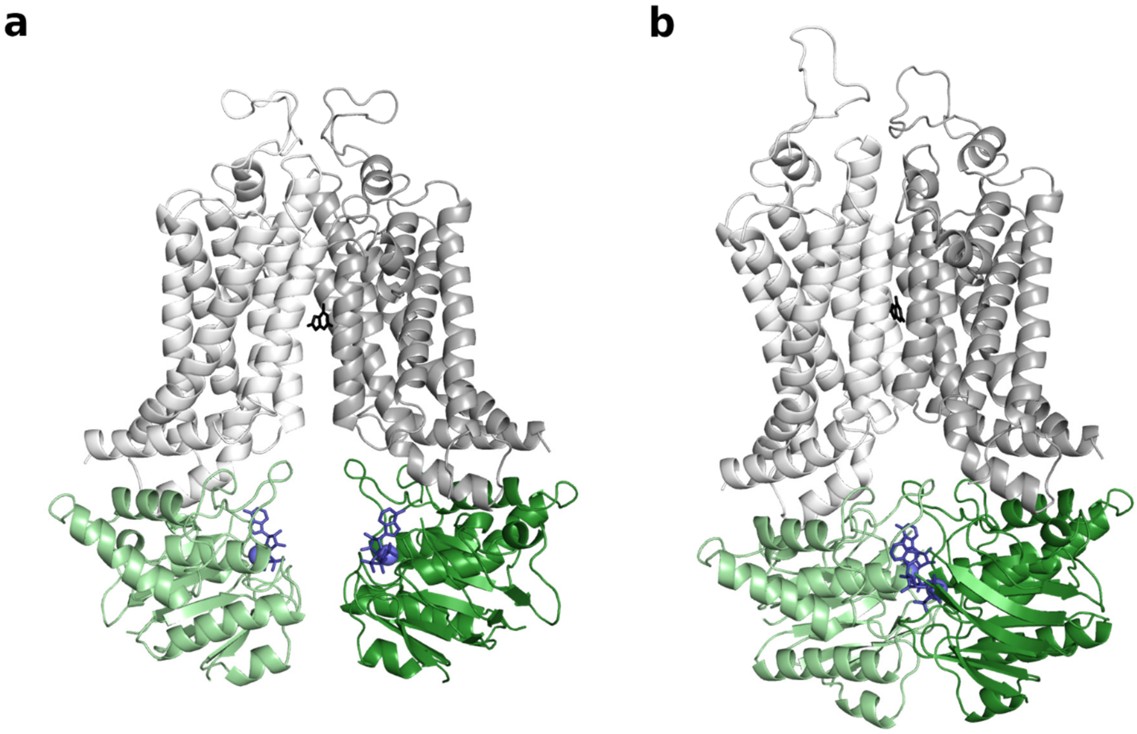

supp_fig_targeted. The 6HCO-based, 6HZM-like, inward-closed conformation with uric acid and ATP was generated in a targeted MD simulation. The inward-facing 6HCO conformation supplemented with two ATP and a uric acid molecules (**a**) was subjected to a targeted MD simulation to generate an ATP bound, 6HZM-like conformation exhibiting closed NBDs and closed intracellular ends of TM helices, with a substrate molecule in the central binding pocket (**b**). Protein Cα and Mg-ATP atoms were used for targeting in GROMACS/PLUMED simulations. Gray: transmembrane domains, green: nucleotide binding domains, blue: Mg-ATP, black: uric acid.

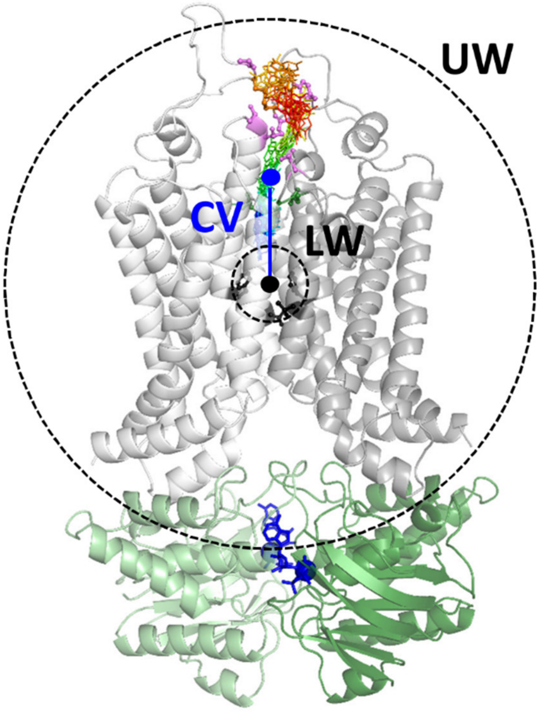

supp_fig_metad. Metadynamics simulation setup. The biased reaction coordinate (collective variable, CV) was defined as the distance (blue line) between the center of mass of four Cα atoms (residues 439 and 542 in both chains, black filled circle) and the center of mass of uric acid (blue filled circle). In order to restrict the movement of uric acid to the final part of the transport pathway, a lower wall (LW) and an upper wall (UW) were defined at 5 Å and 38 Å, respectively (dashed black circles, approximate radii). An exit event is represented by several uric acid molecules (blue to red sticks). Metadynamics simulations were performed using GROMACS/PLUMED.

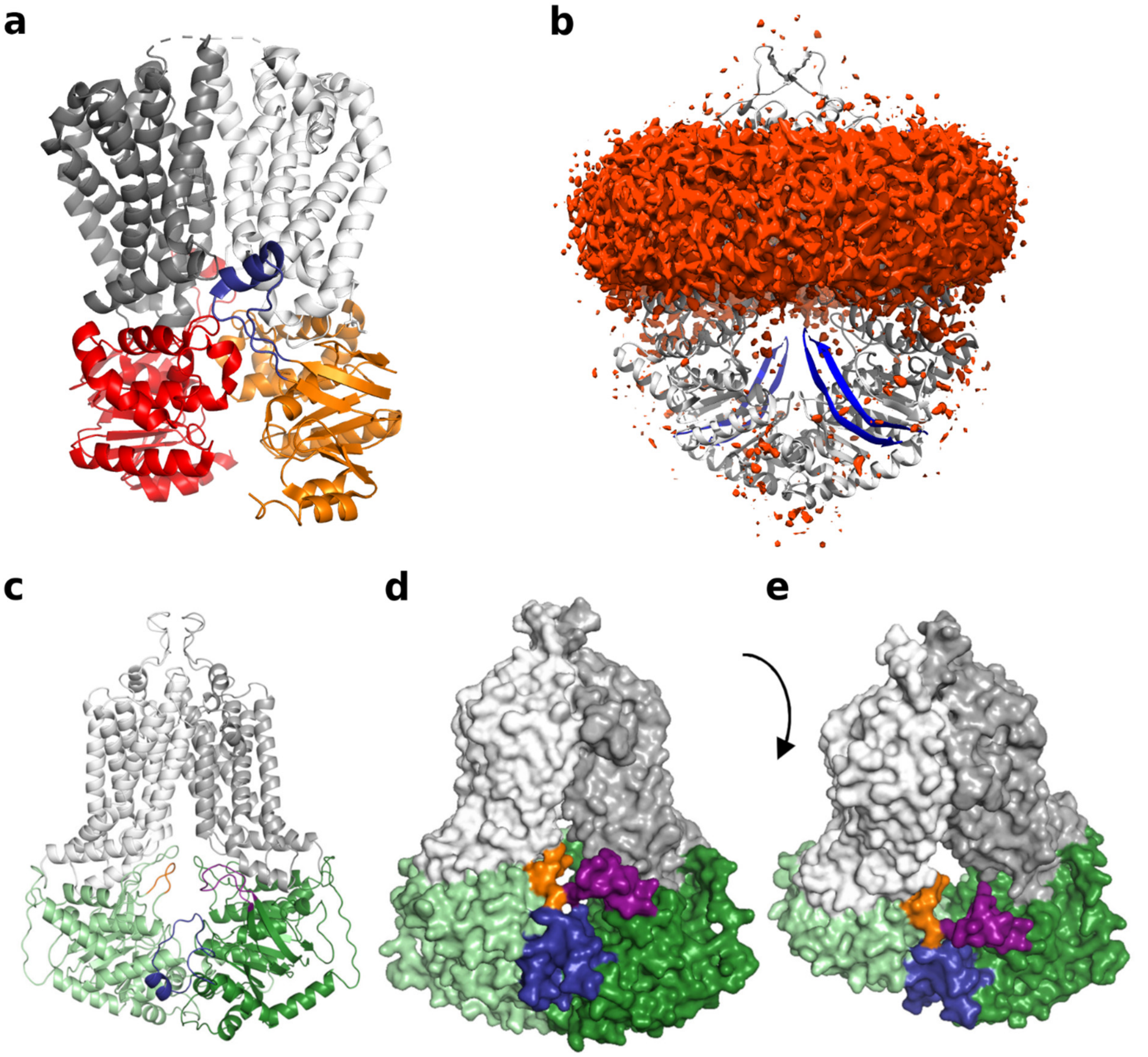

supp_fig_ri. The RI-loop of ABCG2 is membrane bound. (**a**) The O-antigen polysaccharide ABC-transporter (PDB ID: 6OIH), along with other bacterial transporters, contains a loop in its nucleotide binding domain, between the first and second β-strands. This gaiting loop establishes a contact with the lipid bilayer and is important for the first steps of substrate transport, hence its name. Red and orange: nucleotide binding domains, blue: gaiting loop and helix, white and gray: transmembrane domains. (**b**) ABCG2 (PDB ID: 6HIJ, EMD-4256) exhibits a similar loop that is likely to be important for transport and/or cholesterol regulation. Because of spatial similarity to the regulatory insertion of CFTR, this loop is called RI-loop. <u>The loops, unresolved in cryo-EM structures, likely affect the substrate entry.</u> (**c**) Cartoon representation of the ABCG2 structure (PDB ID: 6HIJ) with unresolved loops built using the Modeller software [10]. (**d**) Surface representation of the same structure, which is tilted in (**e**) allowing a top view. The opening behind RI is in the membrane region, thus it is likely to be covered by lipids. Gray and white: transmembrane domains, green and light green: nucleotide binding domains, purple: regulatory insertion, blue: N-terminal part of the linker region, orange: amino acids 177-184, located in a loop of the α-subdomain in the opposite NBD.

The regions at the N- and C-terminal ends of the V-shaped helices in the linker region (L_N and L_C) and the loop between the first two strands of NBD (RI, A-loop) were not resolved

in the current structures [2, 8, 11]. We call the latter loop RI, since it is located at analogous position as the regulatory insertion of CFTR NBD1 [12, 13] and the name A-loop [14] may not be appropriate for this dynamics segment, since it is unlikely to interact with the adenine ring of ATP. Similarly to others [15], we decided to exclude RI and the linker from our simulations, since their absence may provide less uncertainty than the presence of inaccurately modelled segments. Nevertheless, Ferreira *et al.* have modelled the unresolved loops and suggested that the linker region plays an important role in allosteric signal propagation from the NBDs to TMDs, based on principal component analysis [6]. We also initiated the modeling of these loops and test their properties and validity [16]. In our models, L_C was located laterally from the possible entry sites, while residues 310-315 in L_N could influence the entry route, depending on L_N conformation. This region together with RI and amino acids 177-184, located in a loop of the α-subdomain, can significantly reduce the open space towards the inner parts of ABCG2. Therefore, these regions, especially RI may significantly affect substrate access and contribute to substrate specificity. In order to support our hypothesis, we identified variants located within RI, in genetic data (gnomAD) and experimental datasets (http://abcm2.hegelab.org) [17, 18]. The R56Q variant (allele frequency: 4.96E-05, dbSNP: rs543249891 gnomAD ID: 4-89060981-C-T) has been shown to be associated with an increased survival rate in cancer patients treated with doxorubicin [19]. This observation suggests that the R56Q variant is characterized by a decreased efficiency of doxorubicin transport. Ishikawa *et al.* reported a decreased transport of porphyrin and methotrexate by the G51C ABCG2 variant [20, 21]. We identified an additional ABCG2 RI mutant in the literature, namely C55S, which was reported to transport SN-38 similarly to the wild type protein [22]. Based on currently available data we cannot resolve, whether the RI loop in ABCG2 regulates its transport function or it may play a role in cholesterol regulation, since this region most likely interacts with the inner leaflet of the plasma membrane similarly to the loop of the bacterial transporters, including the O-antigen transporter [23-25]. However, the structure of the membrane interacting region cannot be convincingly predicted using bioinformatics tools, as a potential gating helix similar to that in the bacterial transporter.

supp_table_simulations. Summary of simulations.

structure	bilayer composition	n x length	used in
6HIJ	6+4 CHOL, 8 PPE in POPC	3x0.5 µs	Figures 1 and S1
6HCO	POPC	4x1 µs	Figures 2, S3-S6
6HCO	SITO:POPC	4x1 µs	Figures S3-S6
6HCO	CHOL:POPC	4x1 µs	Figures 2, 3, S3-S6
6HZM	CHOL:POPC	2x1 µs	Figure S4 and S6
6HCO, uric acids	CHOL:POPC	3x0.5 µs	Figure 4
6HCO-trp, uric acids	CHOL:POPC	3x1 µs	Text only
6HCO-trp, uric acid	CHOL:POPC	6x(10-30ns)	Figure 5

supp_table_tmends. Intracellular (i) and extracellular (e) ends of TM helices (TH).

**TH#**	
**1**	i 396 415 e
**2**	e 427 445 i
**3**	i 478 498 e
**4**	e 502 527 i
**5**	i 536 556 e
**6**	e 624 643 i

References

1. Laszlo, L., B. Sarkadi, and T. Hegedus, *Jump into a New Fold-A Homology Based Model for the ABCG2/BCRP Multidrug Transporter.* PLoS One, 2016. **11**(10): p. e0164426.
2. Jackson, S.M., et al., *Structural basis of small-molecule inhibition of human multidrug transporter ABCG2.* Nat Struct Mol Biol, 2018. **25**(4): p. 333-340.
3. Corradi, V., et al., *Structure of Transmembrane Helix 8 and Possible Membrane Defects in CFTR.* Biophys J, 2018. **114**(8): p. 1751-1754.
4. Gyimesi, G., et al., *ATP hydrolysis at one of the two sites in ABC transporters initiates transport related conformational transitions.* Biochim Biophys Acta, 2011. **1808**(12): p. 2954-64.
5. Tordai, H., I. Leveles, and T. Hegedus, *Molecular dynamics of the cryo-EM CFTR structure.* Biochem Biophys Res Commun, 2017. **491**(4): p. 986-993.
6. Ferreira, R.J., et al., *Structure-function relationships in ABCG2: insights from molecular dynamics simulations and molecular docking studies.* Sci Rep, 2017. **7**(1): p. 15534.
7. Szollosi, D., et al., *Dissecting the Forces that Dominate Dimerization of the Nucleotide Binding Domains of ABCB1.* Biophys J, 2018. **114**(2): p. 331-342.
8. Manolaridis, I., et al., *Cryo-EM structures of a human ABCG2 mutant trapped in ATP-bound and substrate-bound states.* Nature, 2018. **563**(7731): p. 426-430.
9. Allen, W.J., J.A. Lemkul, and D.R. Bevan, *GridMAT-MD: a grid-based membrane analysis tool for use with molecular dynamics.* J Comput Chem, 2009. **30**(12): p. 1952-8.
10. Fiser, A., R.K. Do, and A. Sali, *Modeling of loops in protein structures.* Protein Sci, 2000. **9**(9): p. 1753-73.
11. Taylor, N.M.I., et al., *Structure of the human multidrug transporter ABCG2.* Nature, 2017. **546**(7659): p. 504-509.
12. Csanady, L., et al., *Functional roles of nonconserved structural segments in CFTR's NH2-terminal nucleotide binding domain.* J Gen Physiol, 2005. **125**(1): p. 43-55.
13. Lewis, H.A., et al., *Structure of nucleotide-binding domain 1 of the cystic fibrosis transmembrane conductance regulator.* EMBO J, 2004. **23**(2): p. 282-93.
14. Ambudkar, S.V., et al., *The A-loop, a novel conserved aromatic acid subdomain upstream of the Walker A motif in ABC transporters, is critical for ATP binding.* FEBS Lett, 2006. **580**(4): p. 1049-55.
15. Khunweeraphong, N., et al., *The ABCG2 multidrug transporter is a pump gated by a valve and an extracellular lid.* Nat Commun, 2019. **10**(1): p. 5433.
16. Mozner, O., et al., *Cellular Processing of the ABCG2 Transporter-Potential Effects on Gout and Drug Metabolism.* Cells, 2019. **8**(10).
17. Gyimesi, G., et al., *ABCMdb: a database for the comparative analysis of protein mutations in ABC transporters, and a potential framework for a general application.* Hum Mutat, 2012. **33**(11): p. 1547-56.
18. Tordai, H., et al., *ABCMdb reloaded: updates on mutations in ATP binding cassette proteins.* Database (Oxford), 2017. **2017**(1).
19. Xiao, Q., et al., *Germline variant burden in multidrug resistance transporters is a therapy-specific predictor of survival in breast cancer patients.* Int J Cancer, 2020. **146**(9): p. 2475-2487.
20. Tamura, A., et al., *Functional validation of the genetic polymorphisms of human ATP-binding cassette (ABC) transporter ABCG2: identification of alleles that are defective in porphyrin transport.* Mol Pharmacol, 2006. **70**(1): p. 287-96.
21. Yoshioka, S., et al., *The identification of two germ-line mutations in the human breast cancer resistance protein gene that result in the expression of a low/non-functional protein.* Pharm Res, 2007. **24**(6): p. 1108-17.
22. Kage, K., T. Fujita, and Y. Sugimoto, *Role of Cys-603 in dimer/oligomer formation of the breast cancer resistance protein BCRP/ABCG2.* Cancer Sci, 2005. **96**(12): p. 866-72.

23. Bi, Y., et al., *Architecture of a channel-forming O-antigen polysaccharide ABC transporter.* Nature, 2018. **553**(7688): p. 361-365.
24. Caffalette, C.A., et al., *A lipid gating mechanism for the channel-forming O antigen ABC transporter.* Nat Commun, 2019. **10**(1): p. 824.
25. Chen, L., et al., *Cryo-electron Microscopy Structure and Transport Mechanism of a Wall Teichoic Acid ABC Transporter.* mBio, 2020. **11**(2).
